# A Comprehensive Systematic Review of Data Linkage Publications on Diabetes in Australia

**DOI:** 10.3389/fpubh.2022.757987

**Published:** 2022-05-25

**Authors:** Ngan T. T. Dinh, Ingrid A. Cox, Barbara de Graaff, Julie A. Campbell, Brian Stokes, Andrew J. Palmer

**Affiliations:** ^1^Health Economics Research Group, Menzies Institute for Medical Research, University of Tasmania, Hobart, TAS, Australia; ^2^Department of Pharmacology, Thai Nguyen University of Medicine and Pharmacy, Thai Nguyen University, Thai Nguyen, Vietnam; ^3^Tasmanian Data Linkage Unit, Menzies Institute for Medical Research, University of Tasmania, Hobart, TAS, Australia; ^4^Centre for Health Policy, School of Population and Global Health, The University of Melbourne, Melbourne, VIC, Australia

**Keywords:** data linkage, record linkage, linked data, linked records, medical record linkage, diabetes, Australia

## Abstract

**Aims:**

Our study aimed to identify the common themes, knowledge gaps and to evaluate the quality of data linkage research on diabetes in Australia.

**Methods:**

This systematic review was developed in accordance with the Preferred Reporting Items for Systematic Reviews and Meta-Analyses (the PRISMA Statement). Six biomedical databases and the Australian Population Health Research Network (PHRN) website were searched. A narrative synthesis was conducted to comprehensively identify the common themes and knowledge gaps. The guidelines for studies involving data linkage were used to appraise methodological quality of included studies.

**Results:**

After screening and hand-searching, 118 studies were included in the final analysis. Data linkage publications confirmed negative health outcomes in people with diabetes, reported risk factors for diabetes and its complications, and found an inverse association between primary care use and hospitalization. Linked data were used to validate data sources and diabetes instruments. There were limited publications investigating healthcare expenditure and adverse drug reactions (ADRs) in people with diabetes. Regarding methodological assessment, important information about the linkage performed was under-reported in included studies.

**Conclusions:**

In the future, more up to date data linkage research addressing costs of diabetes and its complications in a contemporary Australian setting, as well as research assessing ADRs of recently approved antidiabetic medications, are required.

## Introduction

Diabetes mellitus is a chronic disease placing a heavy socioeconomic burden not only on patients and their families, but also on society. In 2017–2018, the National Health Survey reported that 1.2 million Australians were living with diabetes with the rate doubling over the preceding 30 years (1). In addition, type 2 diabetes mellitus (T2DM) was ranked as the 13^th^ leading cause of disease burden in Australia (2). The total healthcare expenditure on people with diabetes was estimated to be A$2.7 billion (2.3% total disease expenditure) in 2015–2016 (3). In this context, the provision of information related to diabetes has been considered to be a national priority (4).

Health data are increasingly stored in large administrative electronic databases (5). Although having been developed primarily for administrative purposes, such as providing billing information and tracking health care reimbursement (6, 7), there has been an increasing trend to use these databases for research purposes based on their specific advantages compared to clinical databases (8). Over time, the need for comprehensive datasets to perform high-quality research led to the development of a novel tool maximizing the usefulness of electronic databases for research: data linkage.

Data linkage, or record linkage as it is also known, is a process that matches records representing the same person or entity derived from different data sources in order to generate new and more comprehensive datasets for different purposes, and particularly research (9). Data linkage has been started since the 1970s in Australia and developed gradually after the establishment of the two first data linkage units: The Western Australian Data Linkage System (WADLS) in 1995, and the Centre for Health Record Linkage (CHeReL) in New South Wales in 2006 (10). These units, especially the WADLS, have contributed to many successful linkage projects that have been considered as necessary information to support policy making. To help realise the potential of data linkage, the Australian Government and state authorities invested approximately A$93 million to establish the PHRN in 2009. The PHRN comprises a network of data linkage units located in each Australian state or territory and a national data linkage unit operated by the Australian Institute of Health and Welfare. After the establishment of the PHRN, the number of data linkage studies has increased substantially, including studies focusing on endocrine disorders, such as diabetes (11, 12).

Data linkage enables researchers to obtain a more comprehensive range of information as the data are collected from different sources (13). More importantly, there is a wide range of data sources from administrative, registry, and clinical databases that have been linked, or can be linked, within states or nationally. However, targeted topics of published data linkage studies have mostly focused on service utilization and disease outcomes (5, 14). Until now, although the potential of data linkage in supporting research on chronic diseases, particularly diabetes is undeniable, data linkage usage, as well as quality of data linkage studies on diabetes, have not been properly examined. This systematic review was performed to synthesize the common themes, knowledge gaps, and to evaluate the quality of data linkage publications on diabetes in Australia.

## Methods

### Information Sources

This systematic review was developed in accordance with the PRISMA Statement (15). The protocol for this review was registered for the International Prospective Register Of Systematic Reviews (PROSPERO, RRID: *SCR019061*) with registration number CRD42020158030.

Using predefined search strategies (Supplementary Appendix 1), the following six key databases were searched to identify relevant articles published until 31st December 2020: MEDLINE (MEDLINE, RRID*: SCR_002185*), EMBASE (EMBASE, RRID: *SCR_001650*), Web of science, Scopus, Econlit, and Google scholar (Google Scholar, RRID: *SCR_008878*). The search strategies were based partly on previous strategies constructed by Tew et al. (11). Given that our systematic review focused on data linkage, we also searched manually for studies published on the PHRN website. To ensure the comprehensiveness of the search, reference lists of included studies and related systematic reviews were scanned to obtain any other relevant studies.

### Study Selection and Eligibility Criteria

All search results were catalogued in EndNote X8 (EndNote, RRID: *SCR_014001*). After removing duplicates, screening and selection of papers were managed in Covidence (Covidence, RRID: *SCR_016484*). We used two screening stages to identify included studies against the inclusion and exclusion criteria: screening of titles and abstracts, and full text screening. Both the first and second screening were performed independently by two reviewers. Prior to formal screening, the review team worked together to screen a small sample of studies to ensure the consistency across reviewers. Discrepancies between reviewers were resolved through discussion to reach consensus.

Studies included in the review satisfied the following criteria:

1. Published in English;
2. Used Australian linked data;
3. Focused on diabetes: primary outcomes directly related to diabetes and/or study population involved people with diabetes.

Studies were excluded if they included at least one of the following:

1. Used linked data from other countries;
2. Included other diseases/health conditions without investigating their relationship with diabetes.
3. Did not report any health outcomes;
4. Did not have full text available;
5. Were duplicate publications; protocols, conference abstracts, case reports, reviews, comments and letters without original data; or animal studies.

### Data Extraction and Methodological Assessment

Data were extracted using a pre-designed form in Microsoft Excel. The form included the following key elements: Author, publication year, jurisdiction(s), data linkage unit, datasets, linkage method, population, sample size, timeframe, study design, diabetes type, main outcomes, main findings, advantages and disadvantages of using linked data reported by authors. One reviewer independently extracted data from included studies, then a second reviewer conducted an audit from a random 10% of papers. This process was also applied for critical appraisal.

The guidelines for studies involving data linkage were used to appraise methodological quality of included studies (16). The guidelines include 14 reporting items belonging to four domains. In order to calculate quality scores, we applied the approach of Patel et al. (17) with modifications. Because each paper used a different number of databases, for the first domain we calculated the average number of points achieved in each item. The total number of points obtained from all items divided by the number of applicable items was the quality score of each study. Categorising studies as “low,” “medium,” and “high” quality was based on the first and third quartiles of quality scores. We calculated Spearman correlation coefficient to assess if there was any trend in quality score over time.

## Results

**Figure 1:**
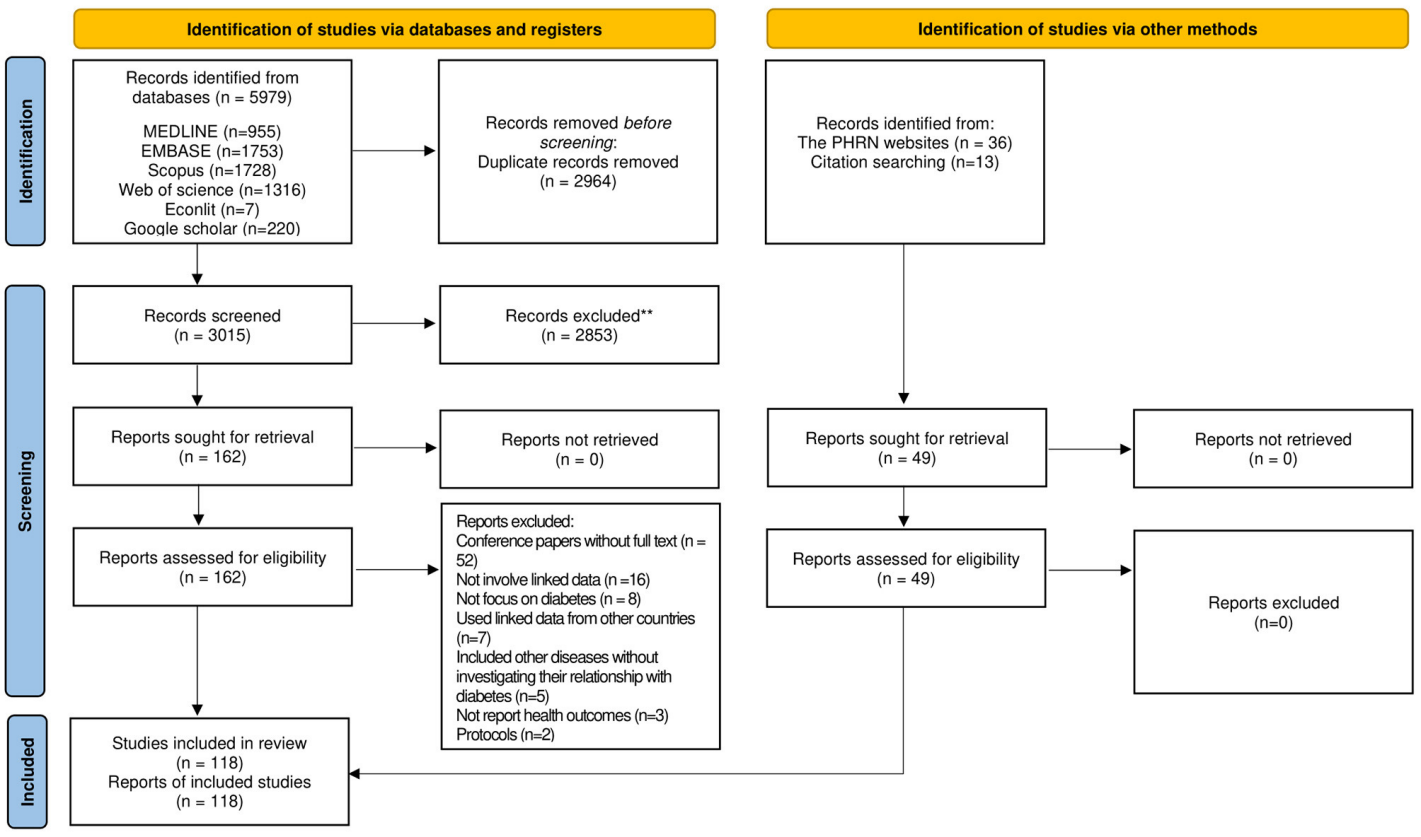
PRISMA 2020 flow diagram for new systematic reviews which included searches of databases, registers and other sources. Figure is adopted from Page et al. (18).

The systematic search identified 5,759 studies. After screening and hand-searching, 118 studies were included in the final analysis (Figure 1). Characteristics of included studies are presented in Supplementary Appendix 2.

### General Finding

#### Jurisdiction of Linkage

**Figure 2:**
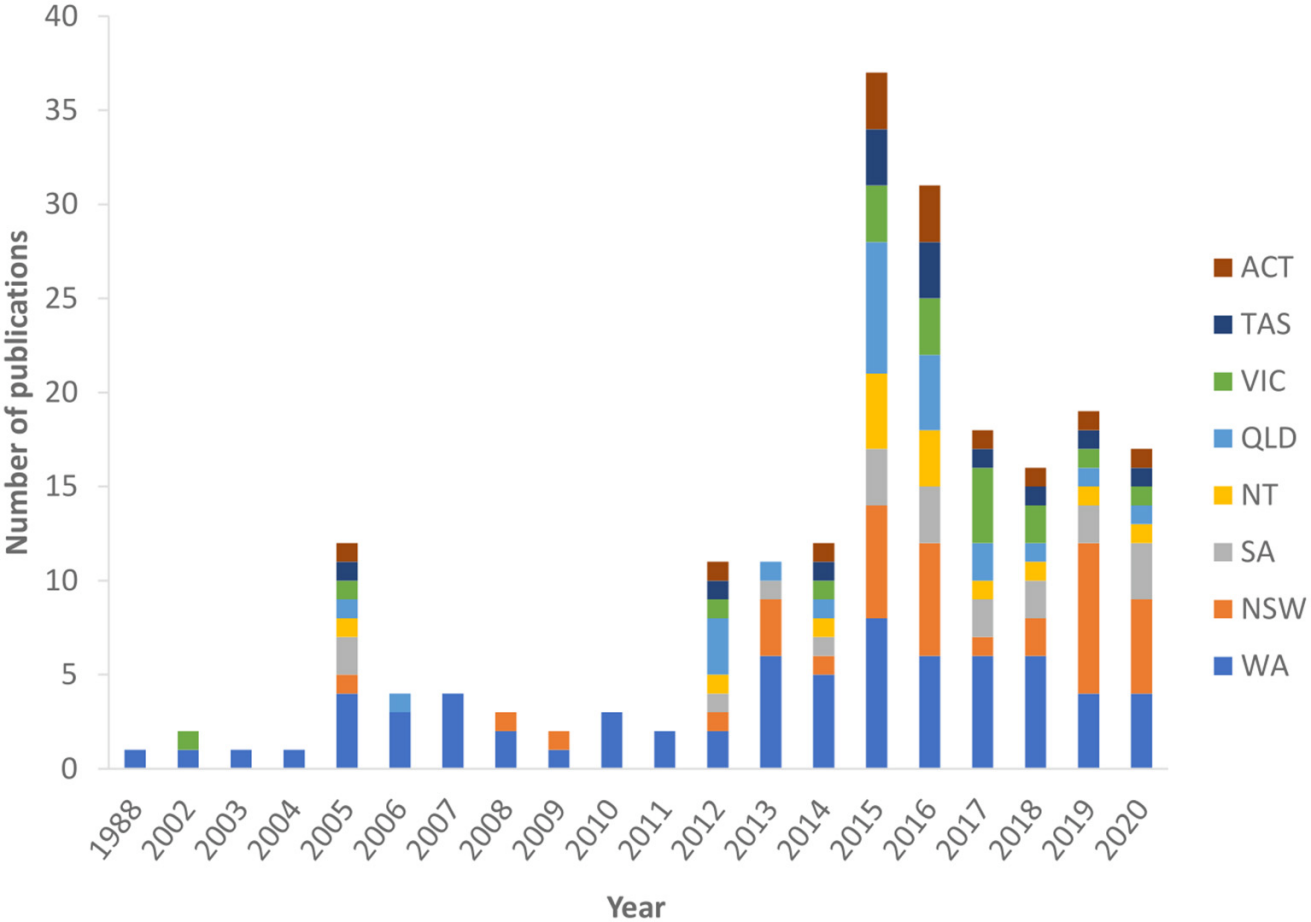
Number of data linkage publications on diabetes by state and publication year. WA, Western Australia; NSW, New South Wales; SA, South Australia; NT, Northern Territory; QLD, Queensland; VIC, Victoria; TAS, Tasmania; ACT, Australian Capital Territory. Studies involving linked data from multiple states were counted as one publication for each state.

The number of data linkage publications on diabetes across Australia has increased over the years, reaching a peak in 2015–2016 and decreasing slightly from 2017 to 2020 (Figure 2). There were 13 national data linkage studies. Studies involving linked data from multiple states were counted as one publication for each state. Most of the contributions (*n* = 108; 51.6%) came from WA (19–90) (*n* = 72; 34.4%) and NSW (72–83, 86, 91–114) (*n* = 36; 17.2%). Studies also originated from QLD (72–83, 86, 115–124) (*n* = 23; 11.0%), SA (72–83, 86, 125–131) (*n* = 20; 9.6%), and VIC (72–83, 86, 129, 132–135) (*n* = 18; 8.6%). A small number of publications were performed in the NT (72–83, 86, 136) (*n* = 14; 6.7%), TAS (72–83) (*n* = 13; 6.2%) (86), and the ACT (72–83) (*n* = 13; 6.2%).

Most included articles (*n* = 77; 65.3%) used data that was linked by a single data linkage organization. However, there were 15 articles (12.7%) using data linked by two organizations. The Western Australian Data Linkage System (WADLS) performed data linkage for nearly half of the included articles (*n* = 53; 44.9%) (19–21, 24–32, 34, 35, 37, 39–61, 63–71, 84, 85, 87–90), 34 articles (28.8%) used data linked by other linkage units belonging to the PHRN network (73–76, 79–81, 86, 91–101, 104, 105, 107–114, 126, 127, 129–131), and only four articles (3.7%) used data linked by non-PHRN units (33, 103, 106, 135). More importantly, whether the linkages were carried out by an organization or researchers themselves, the name of the organization performing the linkage was not explicitly reported in 26 articles (22.0%) (22, 23, 36, 38, 62, 72, 77, 78, 82, 83, 102, 115–125, 128, 132, 134, 136).

#### Method of Linkage and Data Sources

The methods of linkage were not provided in most of the included studies (*n* = 78; 66.1%) (19–21, 23–35, 37, 38, 40–47, 49, 51, 52, 54–59, 61, 63–65, 68–71, 75, 79, 81–83, 85–91, 93, 94, 96–98, 101, 102, 104, 106, 107, 112, 113, 116, 118, 120–122, 124, 125, 127, 128, 133–135). The probabilistic method was predominantly used in the remaining studies (*n* = 31; 26.3%) (22, 36, 39, 48, 50, 53, 60, 62, 66, 67, 72–74, 76, 77, 80, 92, 99, 100, 103, 105, 108, 117, 119, 123, 126, 129–132), while limited studies were undertaken using deterministic linkage (*n* = 2; 1.9%) (115, 136) or a combination of these two methods (*n* = 7; 5.9%) (78, 95, 109–111, 114).

**Figure 3:**
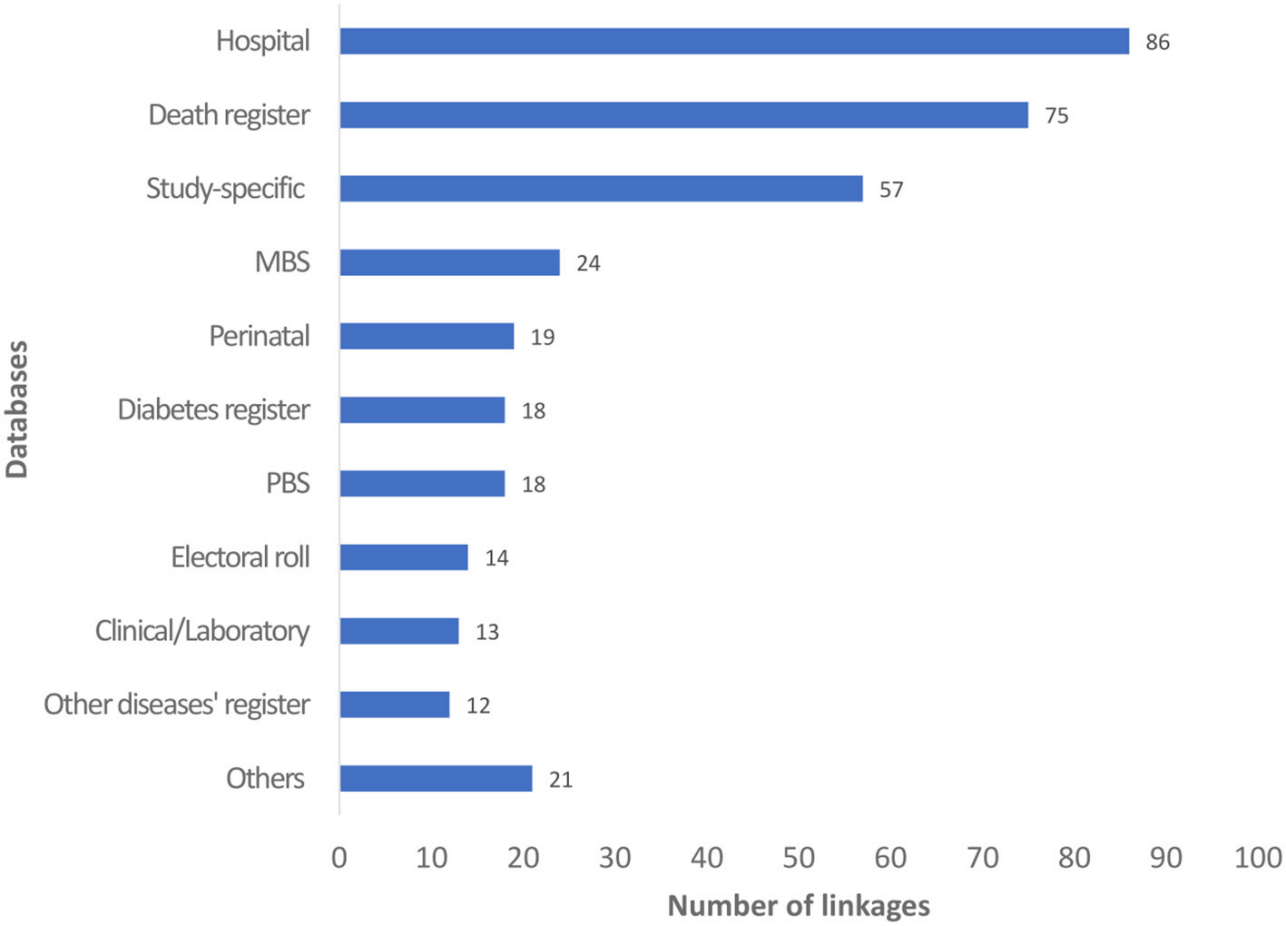
Number of linkages by databases. MBS, Medicare Benefits Schedule; PBS, Pharmaceutical Benefits Scheme.

There were a wide range of Australian datasets linked (Figure 3). Amongst them, the most common databases linked were hospital (*n* = 86; 72.9%), registry of death (*n* = 75; 63.6%) and study-specific databases (*n* = 57; 48.3%). Other commonly linked datasets were the Medicare Benefits Schedule (MBS; *n* = 24; 20.3%), perinatal (*n* = 19; 16.1%); diabetes register (*n* = 18; 15.3%); Pharmaceutical Benefits Scheme (PBS; *n* = 18; 15.3%); electoral roll (*n* = 14; 11.9%); other disease registers (*n* = 12, 10.2%), and clinical/laboratory databases (*n* = 13; 11.0%). Meanwhile, other datasets (*n* = 20; 16.9%), such as medical records, birth register, emergency department, and general practice databases were less common.

#### Timeframe and Diabetes Type

The timeframe of included studies was determined based on the time intervals that data were collected from all linked databases. If the authors did not report their timeframe exactly by date, month and year, the default date of 1^st^ January for the beginning and 31^st^ December for the end of studies was assumed. There were 13 articles (11.0%) that reported a time horizon ≤ 5 years (38, 58, 95, 100, 102, 106, 110, 117, 120, 122, 132, 135, 136). Thirty-five studies (29.7%) used a time frame of 6–10 years (23, 31, 36, 37, 40, 55, 59, 62, 64, 69, 81, 83, 91, 94, 99, 103–105, 107, 109, 111, 115, 116, 118, 119, 121, 123–125, 127, 129, 134). The remaining studies adopted even longer time horizons: 11–15 years (*n* = 37; 31.4%) (20–22, 24, 25, 27, 30, 32, 33, 35, 49, 50, 52, 54, 56, 57, 67, 72– 78, 80, 82, 85, 86, 88, 89, 93, 96, 97, 101, 108, 112, 114, 128, 130), and ≥16 years (*n* = 33; 28.0%) (79).

Diabetes type was ascertained by either International Classification of Diseases (ICD) codes used to identify patients or specific types of diabetes mentioned in the papers. T2DM was investigated in most included papers, either alone (*n* = 44; 37.3%) (19, 20, 24–32, 35, 40, 42–44, 47, 49, 52, 54, 56, 57, 59, 62, 64, 68–71, 75, 85–87, 89–92, 98, 103, 110, 113, 115, 120, 125) or together with other types of diabetes (*n* = 44; 37.3%) (21–23, 34, 36, 37, 39, 41, 46, 48, 51, 58, 61, 63, 65–67, 72–74, 77, 78, 80, 81, 84, 88, 93, 94, 96, 97, 99–101, 104, 106, 109, 111, 114, 117, 123, 124, 127, 128, 136). Fourteen articles (11.9%) focused on type 1 diabetes mellitus (T1DM) (33, 45, 50, 53, 55, 60, 76, 107, 108, 126, 130, 131, 133, 134) and 10 other articles (8.5%) looked at gestational diabetes (GDM) (38, 102, 105, 116, 118, 121, 122, 129, 132, 135). However, there was a small number of papers (*n* = 6; 5.1%) that did not specify the type of diabetes covered (79, 82, 83, 95, 112, 119).

#### Quality of Linked Data

While several measurements can be used to ascertain quality of linkage results, including linkage specificity, sensitivity, false-positive and false-negative rates, most studies reported false-positive and false-negative rates that were relatively low (<1%). However, there were some concerns about missing and incorrect links (23, 67, 129). Other concerns related to the quality of the source information (39, 56, 76, 103, 121, 136), often from administrative databases such as the accuracy of coding (40, 41, 66, 118–120), change in coding system (48, 122), recorder bias (132) or lack of clinical data (21, 22, 34, 45, 46, 72–75, 77, 79, 94, 100, 108, 116, 121, 124, 127).

### Common Themes of Research

There were six common themes of research that emerged amongst publications reviewed in our study. Each of these common themes are discussed in detail below. Because of the large degree of heterogeneity amongst different studies, especially in relation to the target populations and outcomes measurements, we could not perform a meta-analysis.

#### Health Outcomes in People With Diabetes

Nearly half of the included studies (*n* = 40; 33.9%) focused on health outcomes in people with diabetes; in some cases these were examined in the context of other diseases.

##### Publications Investigating Diabetes Alone

Health outcomes targeted were mortality (25, 26, 39, 48, 64, 72, 75–77, 79, 80, 92, 98, 127), hospital admissions (23, 90), and pregnancy outcomes (106, 122, 128, 132) (Supplementary Table 1a-Appendix 2).

These studies found that although there was a downward trend in mortality (72, 77), people with diabetes still had a higher risk of death (39, 48, 79, 127), greater number of years of life lost (127) and shorter life expectancy (76) compared to people without diabetes. In addition, diagnosis at younger ages (72, 75, 92), being Indigenous (25, 98), living in the major urban and remote areas (80) and having complications (such as myocardial infarction and peripheral arterial disease) (54, 64) were some specific factors associated with an increased risk of death from diabetes.

In terms of hospitalization, people with diabetes had higher hospitalization rates for both diabetic and non-diabetic causes compared to the general population (23, 90). Specific to pregnancy outcomes, data linkage studies suggested negative maternal and neonatal outcomes in women having diabetes in pregnancy and their infants such as gestational hypertension, induction of labor, and caesarean section (106, 122, 128, 132).

##### Publications Investigating Diabetes in Relation to Other Diseases or Health Conditions

Because of the higher incidence of some diseases in people with diabetes, association between diabetes and psychiatric disorders (60), dementia (28, 29), infections (43, 119), cancer (42, 73), hip fracture (47), tendon rupture (44), tuberculosis (81), and pancreatitis (41) was reported. There were diseases and health conditions (mental illness, cancer, infection, burn) that solely or in combination with diabetes, were associated with negative outcomes such as increased diabetes-related hospitalization rates (34, 46, 51) or increased mortality (68, 73, 74) (Supplementary Table 1b-Appendix 2).

#### Incidence of Diabetes/Diabetic Complications and Their Risk Factors

There were 11 studies (9.3%) reporting incidence or prevalence of diabetes and its complications, either alone (66, 87, 130) or in combination with investigating predictors (19, 40, 45, 88, 108, 113, 114, 131), and 21 other studies (17.8%) only focused on risk factors. Most of these papers investigated multiple risk factors simultaneously (19, 32, 40, 45, 57, 59, 105, 108, 114, 123, 124). The remaining papers focused specifically on socio-demographic (49), clinical (55, 59, 70, 78, 88, 133), lifestyle (50, 56, 93, 96, 97, 101), or perinatal factors (53, 107, 126) (Supplementary Table 2-Appendix 2).

##### Risk Factors for Diabetes

Results of papers exploring risk factors for T1DM suggested that maternal smoking during pregnancy was associated with lower risk of childhood T1DM (50, 131), but were inconsistent in terms of whether caesarean section (107, 126) and increasing birth weight (53, 107) were determinants of T1DM in children. Papers that investigated the risks associated with T2DM and GDM found that smoking (56, 113), being overweight (56, 113, 124) and using statins (78) were factors associated with T2DM. Having metabolic syndrome pre-pregnancy, or GDM in the previous pregnancy, were strong predictors of GDM (105, 123).

##### Risk Factors for Diabetic Complications

Most of included papers looked at chronic complications, either microvascular (40, 45, 93, 96, 97, 101, 114, 133) or macrovascular complications (19, 49, 55, 57, 59, 70, 85). Only three articles focused on acute complications (32, 88, 108).

In terms of socio-demographic factors, educational status (higher than primary level) (32), ethnicity (Asian or Southern European) (40), sex (women) (45, 108), older age (40, 114) and living in regional or remote areas (108, 114) were associated with some diabetic complications - especially acute and ophthalmic complications. In terms of clinical factors, having other complications (19, 32, 40, 57, 85, 114), longer diabetes duration (32, 40, 133), poor glycaemic (19, 45, 57, 88, 133) and poor lipid control (40, 55) were also strongly associated with increased risk of developing complications, and particularly ophthalmic and foot complications. In terms of lifestyle factors, while physical activity, high consumption of cheese and whole-meal bread showed a positive effect on preventing ophthalmic complications in people with diabetes (96, 97), increasing consumption of red meat and poultry showed harmful effects (101).

#### Validation of Data Sources and Diabetic Instruments

Linked data was used in six articles (5.1%) to validate data accuracy. When cross referenced with other sources, the accuracy of using administrative data to identify diabetes status was relatively high (sensitivity and specificity were both up to 99%) (84, 118). Cross referencing with administrative databases demonstrated that self-reporting of diabetes was a reliable source to identify patients (109). In addition, using linked data was a method which could improve data completeness (33, 103) and reduce bias by supporting exclusion of ineligible subjects (67).

There were 11 studies (9.3%) that successfully developed and/or validated diabetes instruments. These included a new measure of continuity of primary care (21), a stratification strategy to classify diabetes severity (65), a simple instrument to predict vascular disease severity in people with T2DM (115), risk equations to predict life expectancy (62) and mortality (22) for T2DM people after occurrence of major complications, an Australian risk equation to predict CVD for T2DM people (24), the Framingham and United Kingdom Prospective Diabetes Study cardiovascular risk equations (27), three measurement approaches of regularity of general practice (GP) contacts (61), and the International Association of Diabetes Study Groups criteria to diagnose GDM (38, 102, 135).

#### Health Service Utilization in People With Diabetes

Articles that focused on the relationship between primary care and hospitalization (*n* = 6; 5.1%) used different approaches when exploring the concept “continuity of care”. While the two oldest papers only measured the number of GP visits (frequency) (91, 136), more recent publications measured either the dispersion of GP visits over time (regularity) (94) or the combination between regularity and frequency (37, 95, 111). Although most of them suggested the completely inverse association between primary care use and hospitalization (37, 91, 94, 95), two other papers emphasized the importance of maintaining adequate levels of GP contact on reducing hospitalization (111, 136).

The remaining articles (*n* = 8; 6.8%) suggested that people with diabetes exerted a high demand on the health service (83, 100), however discrepancies between Australians and overseas-born Australians (110), between patients with different socioeconomic statuses (86) were noted. The rates of participation in diabetes-related screening, such as postpartum glucose screening after GDM (116, 121, 129) and screening for diabetic retinopathy (112) were relatively low.

#### Intervention and Medications in People With Diabetes

Three studies (2.5%) compared mortality or hospitalization between people with diabetes with and without the interventions to explore their effects. While an integrated primary–secondary model of care were proven to be effective (reduced hospitalization) (120), self-monitoring of blood glucose showed no effects (35).

The effects of medications were investigated in five studies (4.2%). Papers focusing on ADRs of antidiabetic medications found that metformin was associated with hypomagnesemia (71) but was not associated with either lactic acidosis (52) nor adverse maternal and neonatal outcomes (104). In addition, continuous subcutaneous insulin infusion and multiple daily injections showed no difference in adverse pregnancy outcomes (134).

#### Healthcare Costs in People With Diabetes

Seven (5.9%) studies estimated healthcare costs. All costs estimated were direct costs covering several areas of expenditure, but mainly hospital and primary care. The majority were partial economic evaluations (30, 31, 36, 69, 99, 117); only one study performed a cost-effectiveness analysis (125). Overall, healthcare costs in people with diabetes were much higher than people without diabetes (99, 117). Additionally, this expenditure increased exponentially over time because of increasing diabetes prevalence and complications (30, 31, 36, 69).

### Methodological Assessment

**Figure 4:**
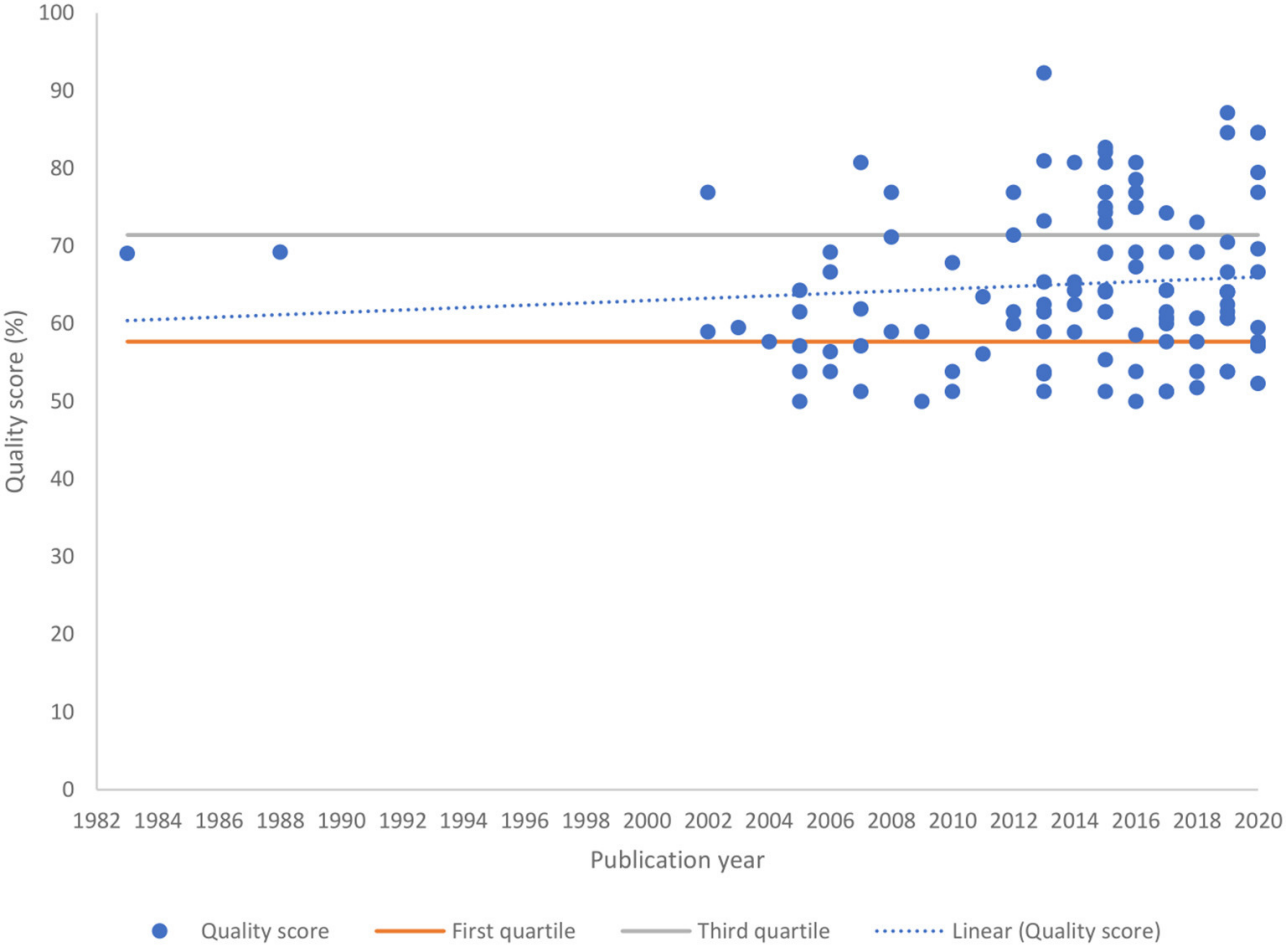
Quality scores of included studies against the guidelines for studies involving data linkage.

After applying the quality assessment guidelines for studies involving data linkage to evaluate the quality of included studies, we identified 32 high-quality, 55 medium-quality and 31 low-quality studies (Figure 4). The Spearman correlation coefficient (0.11) suggested a slightly upward trend in quality scores over time but was not considered statistically significant (*P* = 0.2357).

## Discussion

The results of this review were consistent with previous systematic reviews which showed an upward trend in the number of published papers using Australian linked data over the years, and a variation of research output amongst different states (11, 12). The years 2015 and 2016 saw an expansion in the number of data linkage studies on diabetes in all jurisdictions, and particularly WA and NSW. This could be explained by results from linkage between the National Diabetes Services Scheme (NDSS) and the National Death Index, coinciding with the development of two large data linkage studies: The Fremantle Diabetes Study in WA, and the 45 and Up study in NSW.

There are two main methods used to link data: deterministic and probabilistic matching. Deterministic linkage often requires a set of complete and accurate variables or a unique identifier to create exact matches, whereas probabilistic linkage uses mathematical algorithms to estimate the probability that each pair of records linked refers to the same individual in the population (137). Choosing linkage methods depends on the availability and quality of variables in the datasets. However, with the absence of a unique personal identifier amongst different data sources in Australia (138), it is evident that probabilistic matching was more prevalent.

Databases linked in studies were not restricted to several data sources used commonly in data linkage studies, such as administrative databases [hospital morbidity, death register, Medicare Benefits Schedule (MBS) and Pharmaceutical Benefits Scheme (PBS) claims databases], and register databases [the NDSS, other diseases' registries (tuberculosis, cancer, mental diseases)]. They also included study databases (such as the Fremantle Diabetes, the 45 and Up study's databases) and clinical/laboratory databases. Administrative databases were the sources of information related to mortality, hospital admission and medications. In some studies, linkage containing state electoral roll records or birth registers was performed to identify large comparison cohorts (29, 40–44, 47) or even ascertain migration status of the study population (65). With regard to registry databases, while linkage with the NDSS supported population-based research on diabetes, linkage with other disease registers enabled researchers to investigate the complicated relationship between diabetes and other diseases. Study and clinical/laboratory databases provided researchers with unique variables that were not available in administrative databases including details of lifestyle, and glycaemic and lipid control (HbA1c, HDL cholesterol, LDL cholesterol, triglycerides). Combining all these sources into one comprehensive data set has allowed researchers to answer multiple research questions that would otherwise be difficult to answer from one single source. Based on the diverse information available from linked data, many aspects of diabetes were explored. Data linkage studies supported findings from previous studies with strong evidence based on their large sample sizes, long duration of follow-up, a variety of information related to potential confounders, and outcomes accurately captured from many sources.

### Negative Health Outcomes in People With Diabetes and Their Consequences

In our review, health outcomes in people with diabetes was the most common topic studied. This may be due to the feasibility of ascertaining outcomes data through frequently linked database, such as hospital admission and mortality (12). Another explanation is the potential of linked data to investigate outcomes necessitating prolonged durations to develop, and particularly in chronic diseases such as diabetes.

Data linkage studies found associations between diabetes and negative health outcomes. These included higher risk of death and hospitalization, and higher tendency for negative outcomes in pregnancy. In addition, associations between diabetes and mental illness, cancer, and infections were observed. As a result, demand on health services from people with diabetes was large and the healthcare costs were substantial and increasing exponentially.

### Confirmed and Conflicting Risk Factors for Diabetes and Its Complications

Findings related to risk factors for diabetes and its complications from previous non-data linkage studies have been inconsistent with insufficient power to detect small effects cited as a feature (53). Data linkage studies with community-based samples confirmed the importance of potential risk factors for diabetes (overweight, smoking) and diabetic complications (having other diabetic complications, poor glycaemic and lipid control) reported previously, and suggested conflicting risk factors. These included the association between maternal smoking, caesarean section, increasing birth weight and T1DM in children. This controversial information should be assessed further in future research.

### Data Linkage as a Validation Tool

The potential of linked data as a tool to validate and improve the accuracy of data sources has been mentioned in literature (139, 140). The most frequently linked databases, administrative databases, were the focus of many validation studies using ICD codes (141). More importantly, due to the availability of disparate data sources, data linkage facilitates validation of case-finding algorithms that are constructed using a variety of information, such as a combination of ICD codes and other criteria (MBS and PBS claims, self-reported data…) to identify patients with targeted diseases (141). Apart from studies validating data accuracy, research that was specific to diabetes has been undertaken using linked data to develop and validate diabetes-related instruments which have potential utility in either clinical practice or future research.

### The Association Between Primary Care and Hospitalization

Investigating the association between primary care and hospitalization was very challenging in the past as data are stored in different databases (91). With the development of data linkage methods, it is now possible for researchers to explore this relationship comprehensively. Over time, approaches to measure continuity of primary care have been improved, from monitoring only the frequency of visits to the more recent shift in perspective focusing on the effects of both regularity and frequency of GP visits on hospitalization. Data linkage studies revealed a greater understanding of the connexion between primary care and hospitalization, to help support health policymakers to make evidence-based decisions towards strengthening primary care in diabetes.

### Future Research

The advantages of using linked data to investigate some specific topics, such as healthcare costs and ADRs have been discussed in previous studies (36, 142–145). However, in our review, there were few articles reporting healthcare costs which were either outdated or focused on specific groups which would not provide a comprehensive picture of total costs related to diabetes care. In terms of ADRs, until now, published studies involving linked data have explored ADRs of only a few older antidiabetic medications with well-known ADRs. Given that diabetes imposes a large economic burden on the healthcare system due to its complications and comorbidities, accurately estimating the true economic burden of diabetes is very challenging. In the future, researchers who are interested in data linkage should take into account the availability of linking hospital, study-specific databases, MBS and PBS claims databases to investigate many aspects of healthcare use and expenditure related to diabetes. In addition, assessing ADRs of recently approved medications, such as glucagon-like peptide-1 agonist, dipeptidyl peptidase four inhibitors, and sodium glucose cotransporter 2 should be conducted if there is no profound evidence gathered from existing studies, given the capacity of data linkage to support further examination of the associations between medications and ADRs that were previously established through clinical trials.

In Australia, a systematic review regarding the use of linked hospital data from 1995 onwards identified circulatory diseases, cancer, and mental health diseases as some of the most common topics researched in data linkage studies (11). Similar to diabetes, these diseases could be used as resources for data linkage research.

### Ensuring the Quality of Linked Data

Besides widely recognised advantages listed, there were certain limitations of data linkage that were mostly related to the quality of linked data mentioned above. However, reporting these important details was often ignored in publications. Although some of these limitations are unavoidable, ensuring the quality of linked data is an essential component as it can impact the accuracy and transparency of results (146). Lacking a unique personal identifier amongst different data sources in Australia could be an obstacle for performing linkage with a perfect match. Nevertheless, efforts could be made to increase the quality of linked data, such as developing and opereting a regular quality assurance process for each administrative database.

### Methodological Assessment

There was under-reporting of important information about the linkage, such as methods of linkage, variables used to link data, and quality of linked data. Given the increasing number of data linkage publications, it is important for authors to be careful to report their data transparently and consistently. Adopting guidelines for evaluating the quality of studies using linked data is recommended. However, the quality scores should be interpreted cautiously as some criteria in the guidelines were not applicable for all included studies (16). For example, in the second domain of the guidelines, there is a criterion for reporting changes to coding systems of included datasets; while in some studies no such changes occurred, or the datasets did not require coding. This problem can be resolved by making this criterion optional.

### Strengths and Limitations

This is the first systematic review focusing on data linkage studies on diabetes in Australia. Using search strategies combined with hand searching, we identified 118 relevant studies. Additionally, our study used a new, validated tool designed to appraise the quality of data linkage studies.

However, there were some unavoidable limitations in conducting this review. Firstly, there was potential that the search strategies may have missed relevant studies if studies only described data linkage usage in their full texts, but not in their titles and abstracts. Secondly, this study could not provide further quantitative pooled data, or meta-analysis data to support our findings, due to heterogeneity.

## Conclusion

Our review identified the widespread use of data linkage to address questions related to diabetes in Australia. While some studies have investigated the costs of diabetes and its complications in Australia, more timely research based on data linkage are required to address costs of diabetes and its complications in a contemporary Australian setting. In addition, data linkage studies assessing ADRs of recently approved medications should also be undertaken.

Although we cannot use only evidence gathered from data linkage studies to represent all available data for diabetes, this review will provide a comprehensive picture of what type of evidence that we would expect from data linkage research and whether there are any specific advantages of using data linkage to study diabetes. Findings from this review will contribute to supporting practitioners and policymakers in decision-making and guiding future data linkage research.

## Data Availability Statement

The data that support this study are available in the article and accompanying online Supplementary Material. Further inquiries can be directed to the corresponding author/s.

## Author Contributions

ND conceived the study, performed the literature search, study selection, data extraction and methodological assessment, and prepared the manuscript. IC performed study selection, assisted with data extraction, methodological assessment, and preparation of the manuscript. BdG conceived the study and assisted with preparation of the manuscript. JC conceived the study, assisted with study selection and preparation of the manuscript. BS provided opinion related to data linkage and assisted with preparation of the manuscript. AP conceived the study and assisted with preparation of the manuscript. All authors read and approved the final manuscript.

## Conflict of Interest

The authors declare that the research was conducted in the absence of any commercial or financial relationships that could be construed as a potential conflict of interest.

## Publisher's Note

All claims expressed in this article are solely those of the authors and do not necessarily represent those of their affiliated organizations, or those of the publisher, the editors and the reviewers. Any product that may be evaluated in this article, or claim that may be made by its manufacturer, is not guaranteed or endorsed by the publisher.

## Abbreviations

ADRs: adverse drug reactions
GDM: gestational diabetes
ICD: International Classification of Diseases
MBS: Medicare Benefits Schedule
NDSS: National Diabetes Services
PBS: Pharmaceutical Benefits Scheme
PHRN: Population Health Research Network
T1DM: type 1 diabetes mellitus
T2DM: type 2 diabetes mellitus
WADLS: Western Australian Data Linkage System.
