# Supplementary material for: A Comprehensive Systematic Review of Data Linkage Publications on Diabetes in Australia
Source: Front Public Health. 2022 May 25;10:757987. doi: 10.3389/fpubh.2022.757987 (PMC9174992; doi:10.3389/fpubh.2022.757987)
Supplement: Supplementary file 1 [file Table_1.pdf]

SEARCH STRATEGY

Medline Ovid

#	Search terms	Results
1	(data* and link*).ab,kf,ti.	233095
2	(record* and link*).ab,kf,ti.	43301
3	exp medical record linkage/	4656
4	1 or 2 or 3	257513
5	(Diabetes or Diabetic* or DM or T1D* or T2D* or GDM).ab,kf,ti.	684471
6	exp Diabetes Mellitus/	434241
7	5 or 6	743353
8	(Australia* or AU or New South Wales or NSW or Western Australia or WA or Victoria* or VIC or Tasmania* or TAS or Queensland or QLD or South Australia or SA or Northern Territory or NT or Australian Capital Territory or ACT).ab,kf,ti.	582541
9	exp Australia/	147802
10	Australia.in.	601882
11	8 or 9 or 10	1107583
12	4 and 7 and 11	955

Embase Ovid

#	Search terms	Results
1	(data* and link*).ab,kw,ti.	318230
2	(record* and link*).ab,kw,ti.	62481
3	1 or 2	347871
4	(Diabetes or Diabetic* or DM or T1D* or T2D* or GDM).ab,kw,ti.	1024716
5	exp diabetes mellitus/	993413
6	4 or 5	1219114
7	(Australia* or AU or New South Wales or NSW or Western Australia or WA or Victoria* or VIC or Tasmania* or TAS or Queensland or QLD or South Australia or SA or Northern Territory or NT or Australian Capital Territory or ACT).ab,kw,ti.	729443
8	exp Australia/	171367
9	Australia.in.	944134
10	7 or 8 or 9	1530937
11	3 and 6 and 10	1753

Scopus

#	Search terms	Results
1	TITLE-ABS-KEY (data* AND link*)	633,772
2	TITLE-ABS-KEY (record* AND link*)	94,655
3	#1 OR #2	683,429
4	TITLE-ABS-KEY (Diabetes OR Diabetic* OR DM OR T1D* OR T2D* OR GDM)	1,118,648
5	TITLE-ABS-KEY (Australia* OR AU OR "New South Wales" OR NSW OR "Western Australia" OR WA OR Victoria* OR VIC OR Tasmania* OR TAS OR Queensland OR QLD OR "South Australia" OR SA OR "Northern Territory" OR NT OR "Australian Capital Territory" OR ACT)	2,248,992
6	AFFILCOUNTRY (Australia)	1,965,211
7	#5 OR #6	3,783,018
8	#3 AND #4 AND #7	1,728

Web of science

#	Search terms	Results
1	TOPIC: (data* AND link*)<br>*Indexes=SCI-EXPANDED, SSCI, A&HCI, CPCI-S, CPCI-SSH, ESCI, CCR-EXPANDED, IC Timespan=All years*	411,149
2	TOPIC: (record* AND link*)<br>*Indexes=SCI-EXPANDED, SSCI, A&HCI, CPCI-S, CPCI-SSH, ESCI, CCR-EXPANDED, IC Timespan=All years*	67,588
3	#1 OR #2<br>*Indexes=SCI-EXPANDED, SSCI, A&HCI, CPCI-S, CPCI-SSH, ESCI, CCR-EXPANDED, IC Timespan=All years*	447,156
4	TOPIC: (Diabetes OR Diabetic* OR DM OR T1D* OR T2D* OR GDM)<br>*Indexes=SCI-EXPANDED, SSCI, A&HCI, CPCI-S, CPCI-SSH, ESCI, CCR-EXPANDED, IC Timespan=All years*	865,426
5	TOPIC: (Australia* OR AU OR "New South Wales" OR NSW OR "Western Australia" OR WA OR Victoria* OR VIC OR Tasmania* OR TAS OR Queensland OR QLD OR "South Australia" OR SA OR "Northern Territory" OR NT OR "Australian Capital Territory" OR ACT)<br>*Indexes=SCI-EXPANDED, SSCI, A&HCI, CPCI-S, CPCI-SSH, ESCI, CCR-EXPANDED, IC Timespan=All years*	1,763,671
6	ADDRESS: (Australia)<br>*Indexes=SCI-EXPANDED, SSCI, A&HCI, CPCI-S, CPCI-SSH, ESCI, CCR-EXPANDED, IC Timespan=All years*	1,872,166
7	#5 OR #6	3,305,953

	Indexes=SCI-EXPANDED, SSCI, A&HCI, CPCI-S, CPCI-SSH, ESCI, CCR-EXPANDED, IC Timespan=All years	
8	#3 AND #4 AND #7<br>*Indexes=SCI-EXPANDED, SSCI, A&HCI, CPCI-S, CPCI-SSH, ESCI, CCR-EXPANDED, IC Timespan=All years*	1,316

Econlit

#	Search terms	Search options	Results
S1	(data* AND link*)	Search modes-Boolean/Phrase	25,589
S2	(record* AND link*)	Search modes-Boolean/Phrase	1,421
S3	S1 OR S2	Search modes-Boolean/Phrase	26,248
S4	Australia* OR AU	Search modes-Boolean/Phrase	43,803
S5	"New South Wales" OR NSW OR "Western Australia" OR WA OR Victoria* OR VIC OR Tasmania* OR TAS OR Queensland OR QLD OR "South Australia" OR SA OR "Northern Territory" OR NT OR "Australian Capital Territory" OR ACT	Search modes-Boolean/Phrase	48,945
S6	GE Australia	Search modes-Boolean/Phrase	22,355
S7	S4 OR S5 OR S6	Search modes-Boolean/Phrase	83,718
S8	Diabetes OR Diabetic* OR DM OR T1D* OR T2D* OR GDM	Search modes-Boolean/Phrase	1,653
S9	S3 AND S7 AND S8		7

Google scholar: (data OR record) link diabetes Australia (17,500 results)
PHRN website: 36
