# Supplementary material for: A Comprehensive Systematic Review of Data Linkage Publications on Diabetes in Australia
Source: Front Public Health. 2022 May 25;10:757987. doi: 10.3389/fpubh.2022.757987 (PMC9174992; doi:10.3389/fpubh.2022.757987)
Supplement: Supplementary file 2 [file Table_2.pdf]

Appendix. Characteristics of included studies (n=118)

Study	Quality score	Publication year	Jurisdiction(s)	Data linkage unit	Linkage method	Population	Diabetes type	Study design	Theme
Categorised high quality (n=32); quality score ≥ 71%									
Elizabeth Jean Comino (1)	92%	2013	NSW	CHeReL, Department of Human Services	Deterministic, Probabilistic	Diabetes/non-diabetes/diabetes uncertain	1, 2, GDM	Prospective cohort	3
M. Begum (2)	87%	2019	SA	SA NT Datalink	Probabilistic	Children (vaginal delivery/caesarean)	1	Retrospective population-based cohort	2
Sarah Dennis (3)	85%	2019	NSW	The UNSW electronic Practice Based Research Network	Probabilistic	People with diabetes	2	Retrospective cohort	3
Mumtaz Begum (4)	85%	2020	SA	SA NT datalink	Probabilistic	People with diabetes, with/without maternal smoking	1	Retrospective cohort	2
Ninh Thi Ha (5)	85%	2020	NSW	CHeRel, the Sax Institute	Deterministic, Probabilistic	People with diabetes	1, 2	Retrospective cohort	4
Elizabeth Jean Comino (6)	83%	2015	NSW	CHeReL, the Sax Institute	Deterministic, Probabilistic	People with diabetes		Prospective cohort	4
Lee Nedkoff (7)	81%	2013	WA	WADLS	Deterministic, Probabilistic	CHD patients diagnosed by ICD-9/ICD-10	1, 2	Retrospective cohort	3
Julia R. Kurowski (8)	82%	2015	WA	WADLS	Probabilistic	People with diabetes /CVD people without diabetes	1, 2	Retrospective cohort	2

Jessica L. Harding (9)	81%	2016	NSW, WA, SA, NT, ACT, TAS, QLD, VIC		Probabilistic	People with diabetes	1, 2	Prospective population-based cohort	1
Dianna Josephine Magliano (10)	81%	2015	NSW, WA, SA, NT, ACT, TAS, QLD, VIC	AIHW	Probabilistic	People with diabetes	1, 2	Prospective population-based cohort	1
Jessica L. Harding (11)	81%	2014	NSW, WA, SA, NT, ACT, TAS, QLD, VIC		Probabilistic	People with diabetes	1, 2	Prospective population-based cohort	1
A. Haynes (12)	81%	2007	WA	WADLS	Probabilistic	People with diabetes	1	Prospective population-based cohort	2
Jedidiah I. Morton (13)	79%	2020	NSW, WA, SA, NT, ACT, TAS, QLD, VIC	AIHW		People with diabetes	2	Prospective cohort	4
Lee Nedkoff (14)	79%	2016	WA	WADLS	Probabilistic	Incident MI cases with/without diabetes	1, 2	Retrospective population-based cohort	1
Odette R Gibson (15)	77%	2012	QLD		Deterministic	Indigenous people with diabetes	2	Retrospective/ Prospective cohort	3
Jessica L. Harding (16)	77%	2015	NSW, WA, SA, NT, ACT, TAS, QLD, VIC	AIHW	Probabilistic	People with diabetes	1, 2	Prospective population-based cohort	1

Christine A Stone (17)	77%	2002	VIC		Probabilistic	Pregnant women with/without GDM	GDM	Population-based cross-sectional	1
Jennifer Stewart Williams (18)	77%	2016	NSW	CheReL	Probabilistic	Mid-aged women with/without diabetes	1, 2	Prospective cohort	6
A. W. Shand (19)	77%	2008	NSW	The NSW Department of Health		Pregnant women with/without GDM	1, 2, GDM	Prospective population-based cohort	1
Yuejen Zhao (20)	77%	2015	NT		Deterministic	Indigenous patients	1, 2	Retrospective cohort	4
Mumtaz Begum (21)	77%	2020	SA	SA NT datalink	Probabilistic	Children	1	Cross-sectional	2
Abdulghani H. Al-Saeed (22)	75%	2016	NSW	AIHW	Probabilistic	People with diabetes	2	Prospective cohort	1
Turki J. Alharbi (23)	75%	2015	NSW	AIHW		People with diabetes	2	Retrospective cohort	1
Ming Li (24)	75%	2016	QLD		Probabilistic	Indigenous patients with/without diabetes		Prospective cohort	1
Elizabeth Jean Comino (25)	74%	2015	NSW	CHeReL	Probabilistic	People with/without diabetes	1, 2	Prospective population-based cohort	4
M. N. Cooper (26)	74%	2017	WA	WADLS	Probabilistic	People with/without diabetes	1	Retrospective population-based cohort	1

Alison J. Hayes (27)	73%	2013	WA		Probabilistic	People with diabetes	1, 2	Retrospective population-based cohort	3
J. Zhang (28)	73%	2015	QLD			People with diabetes, with/without the integrated model of care	2	Prospective controlled trial	5
David Youens (29)	73%	2018	WA	WADLS	Probabilistic	People with diabetes, people at high risk of diabetes/CVD	1, 2	Retrospective population-based cohort	3
Sandra K. Campbell (30)	71%	2012	QLD		Probabilistic	Indigenous pregnant women	1, 2, GDM	Prospective cohort	2
Philip Clarke (31)	71%	2008	WA		Probabilistic	People with diabetes	1, 2	Retrospective population-based cohort	6
Rachael E Moorin (32)	71%	2019	NSW	CHeReL, the Sax Institute		People with diabetes	1, 2	Cross-sectional	4
Categorised moderate quality (n = 55); quality score 59–70 %									
Timothy M E Davis (33)	70%	2020	WA	WADLS		People with diabetes	1, 2	Prospective cohort	2
Christopher Glatthaar (34)	69%	1988	WA	The Health Department of Western Australia		Children	1	Population-based cross-sectional/case-control	3
Philip Clarke (35)	69%	2006	QLD		Probabilistic	People with/without diabetes	1, 2	Case-control	6

Lili Huo (36)	69%	2018	NSW, WA, SA, NT, ACT, TAS, QLD, VIC	AIHW	Probabilistic	People with diabetes	2	Prospective population-based cohort	1
Lili Huo (37)	69%	2016	NSW, WA, SA, NT, ACT, TAS, QLD, VIC	AIHW	Probabilistic	People with diabetes	1	Prospective population-based cohort	1
Mark Jones (38)	69%	2017	NSW, WA, SA, NT, ACT, TAS, QLD, VIC		Deterministic, probabilistic	Elderly women with /without statin use	2, GDM	Prospective population-based cohort	2
D.J. Magliano (39)	69%	2015	NSW, WA, SA, NT, ACT, TAS, QLD, VIC	AIHW	Probabilistic	People with diabetes	1, 2	Retrospective cohort	1
Douglas I. R. Boyle (40)	69%	2018	VIC, SA	SA NT Datalink, Victorian Data linkage unit	Probabilistic	Pregnant women	GDM	Retrospective population-based cohort	4
Lee Nedkoff (41)	69%	2015	WA	WADLS	Probabilistic	Incident MI cases with/without diabetes	1, 2	Retrospective population-based cohort	1
Nicholas H De Klerk (42)	69%	1983	WA			People with diabetes	1, 2	Retrospective population-based cohort	1
Timothy M. E. Davis (43)	68%	2010	WA	WADLS		People with diabetes	2	Prospective cohort	2
Duong Thuy Tran (44)	67%	2016	NSW	CHeRel, the Sax Institute	Deterministic, Probabilistic	People with diabetes	2	Prospective cohort	4

Xixi Yan (45)	67%	2019	NSW	the Sax Institute		People with diabetes	1, 2	Prospective cohort	2
W. A. Davis (46)	67%	2006	WA	WADLS		People with diabetes	2	Prospective cohort	2
Louisa Sukkar (47)	67%	2020	NSW	CHeRel, the Sax Institute	Deterministic, Probabilistic	People with diabetes	1, 2	Prospective cohort	2
Aveni Haynes (48)	65%	2014	WA	WADLS	Probabilistic	Children whose mother smoked/did not smoke during pregnancy	1	Prospective population-based cohort	2
A. Z. Khambalia (49)	65%	2013	NSW	CHeReL	Probabilistic	Pregnant women	GDM	Prospective population-based cohort	2
Mendel Baba (50)	64%	2014	WA	WADLS		People with diabetes	2	Prospective cohort	2
Wendy A. Davis (51)	64%	2005	WA	WADLS		People with diabetes	2	Prospective cohort	6
Matthew N. Cooper (52)	64%	2017	WA	WADLS		People with diabetes /general population sample	1	Prospective population-based cohort	2
Catherine R. Chamberlain (53)	64%	2017	QLD			Infants of mothers with/without GDM	GDM	Retrospective cohort	1
Julie Hart (54)	64%	2015	WA	WADLS		People with diabetes	1, 2	Prospective cohort	1
Xiaotong Han (55)	64%	2019	NSW	The Sax Institute		People with diabetes	1, 2	Prospective cohort	2
Changfan Wu (56)	64%	2019	NSW	The Sax Institute		People with diabetes	1, 2	Retrospective cohort	2

Catherine R. Chamberlain (57)	64%	2015	QLD			Indigenous/non-indigenous pregnant women	2, GDM	Retrospective population-based cohort	2
Amanda Ampt (58)	64%	2019	NSW	CHeReL	Probabilistic	People with diabetes	1	Retrospective population-based cohort	2
A. J. Hayes (59)	63%	2011	WA		Probabilistic	People with diabetes	2	Retrospective population-based cohort	3
T. M. E. Davis (60)	63%	2014	WA	WADLS		People with/without diabetes	1, 2	Population-based case-control	1
Emma J. Hamilton (61)	63%	2013	WA	WADLS		People with/without diabetes	2	Prospective cohort	1
David Youens (62)	63%	2019	WA	WADLS, commonwealth Department of Health and Ageing		Patients with confirmed diabetes or likely to have diabetes	1, 2	Retrospective population-based cohort	3
W. A. Davis (63)	62%	2007	WA	WADLS		People with diabetes, with/without SMBG	2	Prospective cohort	5
D. G. Bruce (64)	62%	2005	WA	WADLS		People with diabetes, with/without depression	2	Prospective cohort	1
Catherine Chamberlain (65)	62%	2015	QLD			Indigenous/Non-Indigenous women with GDM	GDM	Retrospective cohort	4

Wendy A. Davis (66)	62%	2013	WA	WADLS	People with diabetes	2	Prospective cohort	6
Catherine Chamberlain (67)	62%	2013	QLD		Pregnant Women with GDM	GDM	Retrospective cohort	3
Changfan Wu (68)	62%	2019	NSW	the Sax Institute	People with diabetes	1, 2	Prospective cohort	2
Yasmine Ali Abdelhamid (69)	62%	2017	SA	AIHW	People with/without diabetes	1, 2	Retrospective cohort	1
Catherine Chamberlain (70)	62%	2015	QLD		Indigenous/Non-Indigenous women with GDM	GDM	Retrospective cohort	4
Claudia Caroline Dobler (71)	62%	2012	NSW, WA, SA, NT, ACT, TAS, QLD, VIC	AIHW	Australians	1, 2	Retrospective population-based cohort	1
Ninh Thi Ha (72)	61%	2019	WA	WADLS, Commonwealth Department of Health and Ageing	People with diabetes	1, 2	Retrospective population-based cohort	3
David G. Bruce (73)	61%	2019	WA	WADLS	People with diabetes	2	Prospective cohort	1
Ninh Thi Ha (74)	61%	2018	WA	WADLS, Commonwealth Department of Health and Ageing	People with diabetes	1, 2	Retrospective population-based cohort	4

Jocelyn J. Drinkwater (75)	61%	2018	WA	WADLS	People with/without diabetes	2	Prospective cohort	2
Emma Hamilton (76)	61%	2017	WA	WADLS	People with/without diabetes	2	Prospective cohort	1
Ninh Thi Ha (77)	61%	2017	WA	WADLS, Commonwealth Department of Health and Ageing	People with diabetes	1, 2	Retrospective population-based cohort	3
Wendy A. Davis (78)	60%	2017	WA	WADLS	People with/without diabetes	2	Prospective cohort	1
Dianna J Magliano (79)	60%	2012	WA	WADLS	People with/without diabetes	2	Prospective cohort	1
Melissa Gillett (80)	60%	2003	WA	WADLS	People with diabetes	2	Prospective cohort	2
Alice A. Gibson (81)	60%	2020	NSW	CHeReL, Department of Human Services	People with diabetes		Prospective cohort	4
Wendy A Davis (82)	59%	2009	WA	WADLS	People with diabetes	2	Prospective cohort	3
Kate J Brameld (83)	59%	2002	WA	WADLS	People with diabetes	2	Retrospective cohort	1
H. Haji Ali Afzali (84)	59%	2013	SA		People with diabetes in high-level/low level model of care	2	Retrospective cohort	6

Niklaus Kamber (85)	59%	2008	WA	WADLS	People with diabetes treated with/without metformin	2	Prospective cohort	5
M. H. B. Zakaria (86)	59%	2014	WA	WADLS	People with/without diabetes	2	Population-based Case-control	1
Janine M. Duke (87)	59%	2016	WA	WADLS	Burn patients/non-injury people	1, 2	Retrospective population-based cohort	1
Categorised low quality (n = 31); quality score ≤ 58 %								
N. Wah Cheung (88)	58%	2018	NSW		Pregnant women with/without GDM	GDM	Retrospective cohort	3
T. M. E. Davis (89)	58%	2004	WA	WADLS	People with diabetes	2	Prospective cohort	1
Sally K. Abell (90)	58%	2017	VIC	Monash Centre for Health Research and Implementation	Pregnant women with/without GDM	GDM	Retrospective population-based cohort	3
Timothy M. E. Davis (91)	58%	2012	WA	WADLS	Aboriginal/Anglo-Celt people with diabetes	2	Prospective cohort	2
Phillipa B. Sharpe (92)	57%	2005	SA		Infants of mother with pre-existing DM/GDM/ IGT	1, 2, GDM	Retrospective population-based cohort	1
Valerie Burke (93)	57%	2007	WA	WADLS	Aboriginal people	2	Prospective cohort	2
Jocelyn J. Drinkwater (94)	57%	2020	WA	WADLS	People with diabetes	2	Prospective cohort	2

Timothy M.E. Davis (95)	57%	2020	WA	WADLS	People with diabetes	2	Prospective cohort	5
Paul E. Norman (96)	56%	2006	WA	WADLS	People with diabetes	2	Prospective cohort	1
Qun Mai (97)	56%	2011	WA	WADLS	MHCs/Non-MHCs	1, 2	Retrospective population-based cohort	1
Wendy Angela Davis (98)	55%	2015	WA	WADLS	People with/without diabetes	2	Prospective cohort	1
Erin Kelty (99)	54%	2019	NSW	CHeReL, AIHW	Pregnant women using gliclazide/metformin	2, GDM	Retrospective cohort	5
David G. Bruce (100)	54%	2010	WA	WADLS	People with diabetes	2	Prospective cohort	2
Befikadu L. Wubishet (101)	54%	2019	NSW, WA, SA, NT, ACT, TAS, QLD, VIC	AIHW	Women with prevalent/incident diabetes/without diabetes		Prospective cohort	1
W. A. Davis (102)	54%	2006	WA	WADLS	People with diabetes	2	Prospective cohort	6
Kirsten E. Peters (103)	54%	2018	WA	WADLS	People with diabetes	2	Prospective cohort	2
Kirsten E. Peters (104)	54%	2013	WA	WADLS	People with diabetes	2	Prospective cohort	5
James Tatoulis (105)	54%	2016	NSW, WA, SA, NT,		Patients underwent coronary artery bypass surgery		Retrospective cohort	5

			ACT, TAS, QLD, VIC					
A.F. Young (106)	54%	2005	NSW, WA, SA, NT, ACT, TAS, QLD, VIC		Mid-aged and older women with/without diabetes		Prospective cohort	4
Elizabeth J. Comino (107)	54%	2013	NSW	CHeReL	People with diabetes	2	Retrospective cohort	4
Hongmei Zhang (108)	52%	2020	NSW	CHeRel, the Sax Institute	Australian adults	2	Prospective cohort	2
Janine M. Duke (109)	52%	2018	WA	WADLS	Burn/non-burn trauma /non-injured people	1, 2	Retrospective population-based cohort	1
W. A. Davis (110)	51%	2010	WA	WADLS	People with diabetes	2	Prospective cohort	3
T. M. E. Davis (111)	51%	2007	WA	WADLS	Aboriginal/Anglo-Celt people with diabetes	2	Prospective cohort	1
E. D. D. Tan (112)	51%	2013	WA	WADLS	Asian/Anglo-Celt people with diabetes	2	Prospective cohort	1
Mary White (113)	51%	2017	VIC	BioGrid	People with diabetes	1	Retrospective cohort	2
Sally K. Abell (114)	51%	2017	VIC		Pregnant women treated with CSII/MDI	1	Retrospective cohort	5
W. A. Davis (115)	51%	2015	WA	WADLS	People with/without diabetes	1, 2	Prospective cohort	1

Aminath LAAFIRA (116)	50%	2016	WA		Pregnant women with/without GDM	GDM	Retrospective cohort	3
C. S. Algert (117)	50%	2009	NSW	CHeReL	People with diabetes	1	Prospective cohort	2
T. M. E. Davis (118)	50%	2005	WA	WADLS	People with diabetes	1	Prospective cohort	2

ACT Australian Capital Territory, *AIHW* Australian Institute of Health and Welfare, *CHD* Coronary heart disease, *CHeReL* Centre for Health Record Linkage, *CSII/MDI* continuous subcutaneous insulin infusion/multiple daily injections, *CVD* Cardiovascular disease, *DM* Diabetes Mellitus, *GDM* Gestational diabetes, *IGT* Impaired glucose tolerance, *MHCs* Mental Health Clients, *MI* Myocardial infarction, *NSW* New South Wales, *NT* Northern Territory, *QLD* Queensland, *SA* South Australia, *SMBG* Self-monitoring of blood glucose, *TAS* Tasmania, *VIC* Victoria, *WA* Western Australia, *WADLS* Western Australian Data Linkage System

Theme 1 Health outcomes in people with diabetes
Theme 2 Incidence of diabetes/diabetic complications and their risk factors
Theme 3 Validation of data sources and diabetic instruments
Theme 4 Health service utilisation in people with diabetes
Theme 5 Intervention and medications in people with diabetes
Theme 6 Healthcare costs in people with diabetes

Table 1a: Detailed characteristics of studies investigating health outcomes in people with diabetes

Study	Target group	Comparator	Cause of death/hospitalisation	Outcome	Main outcome measure	Result	95% CI
Nicholas H De Klerk (42)	People with diabetes	General population	Road trauma	Hospitalisation	Number of hospitalisations	Diabetic men aged under 55:<br>36 observed, 21.2 expected	
Kate J Brameld (83)	People with diabetes		Diabetes	Hospitalisation	Hospitalisation rates	35/100 PY	
Jessica L. Harding (9)	People with diabetes		All cause*, CVD, diabetes, cancer	Mortality	Age-standardised mortality rates	T1DM: decreased 0.61 per 1000 PY<br>T2DM: decreased 0.18 per 1000 PY	-0.91, -0.31<br>-0.25, -0.11
Abdulghani H. Al-Saeed (22)	People with diabetes, age of onset 15-29	General population	All cause	Mortality	SMRs	3.4	2.7-4.2
T. M. E. Davis (111)	Aboriginal people with diabetes	Anglo-Celt people with diabetes	All cause	Mortality	HR	2.37	1.06-5.29
E. D. D. Tan (112)	Asian people with diabetes	Anglo-Celt people with diabetes	All cause,<br>CVD	Mortality	HR	0.58<br>0.13	0.31–1.10<br>0.02-0.96
Turki J. Alharbi (23)	Indigenous/<br>Pacific Islander/<br>Mediterranean/<br>Arabic/<br>Indian/<br>Chinese people with diabetes	Anglo-Celt people with diabetes	All cause*, CVD, renal disease	Mortality	HR	2.3<br>1<br>0.8<br>0.7<br>0.6<br>0.4	1.7-3.0<br>0.8-1.3<br>0.7-0.9<br>0.6-0.8<br>0.5-0.8<br>0.4-0.5

Lili Huo (36)	People with diabetes	People with diabetes diagnosed 10 years older	All cause*, CVD, cancer	Mortality	HR	1.3	
Lee Nedkoff (41)	Incident MI cases with diabetes	Incident MI cases without diabetes	CVD, diabetes	Mortality	OR	1998-2001: 1.23<br>2008-2010: 0.64	1.01-1.5<br>0.46-0.88
Lili Huo (37)	People with diabetes	General population	All cause	Mortality	Life expectancy	-12.2	-11.8, -12.7
Lee Nedkoff (14)	Incident MI cases with diabetes	Incident MI cases without diabetes	All cause*, CVD	Mortality	HR	2006-2009<br>Men: 1.31<br>Women: 1.43	1.13-1.53<br>1.17-1.74
Yasmine Ali Abdelhamid (69)	People with diabetes	People without diabetes	All cause	Mortality	HR	1.16	1.10-1.21
Jessica L. Harding (11)	People with diabetes		All cause*, CVD	Mortality	SMRs	T1DM:<br>Males 4.2 (1997), 3.08 (2010);<br>Females 3.92 (1997), 3.46 (2010)<br>T2DM:<br>Males 1.4 (1997), 1.21 (2010)<br>Females 1.56 (1997), 1.22 (2010)	3.66–4.82;<br>2.77–3.42<br>3.19–4.82;<br>3.01–3.97<br>1.36–1.44;<br>1.19–1.23<br>1.51–1.61;<br>1.19–1.24
Paul E. Norman (96)	People with diabetes and PAD	People with diabetes, without PAD	CVD	Mortality	HR	1.67	1.13-2.47
Befikadu L.	Women with prevalent/incident diabetes	Women without diabetes	All cause	Mortality	HR	1.73<br>1.3	1.57-1.92<br>1.16-1.45

Wubishet (101)							
D.J. Magliano (39)	People with diabetes in major, inner, outer and remote areas		All cause*, CVD, diabetes	Mortality	SMRs	T1DM, Males/Females<br>Major: 3.53/ 4.10<br>Inner: 3.29/ 3.53<br>Outer: 3.11/ 4.79<br>Remote: 3.57/ 5.33<br>T2DM, Males/Females<br>Major: 1.29/ 1.32<br>Inner: 1.21/ 1.29<br>Outer: 1.20/ 1.23<br>Remote: 1.33/ 1.4	3.38 –3.69; 3.87–4.33<br>3.06–3.54; 3.19–3.91<br>2.79–3.46; 4.22–5.43<br>2.92–4.36; 4.32–6.58<br>1.28 –1.30; 1.31– 1.33<br>1.19 –1.22; 1.21–1.25<br>1.18–1.22; 1.20–1.26<br>1.28–1.40; 1.33–1.47
T. M. E. Davis (89)	People with diabetes with silent MI (2)/self-reported CHD no Q waves (3)/self-reported CHD with Q waves (4)	People with diabetes, without MI (1)	All cause*, CVD	Mortality	HR	(2): 1.09<br>(3): 1.91<br>(4): 2.32	0.58-2.03<br>1.45-2.50<br>1.47-3.65
Christine A Stone (17)	Pregnant women with GDM	Pregnant women without GDM		Pregnancy outcomes	OR	Hypertension/pre-eclampsia: 1.6<br>Hyaline membrane disease: 1.6<br>Neonatal jaundice: 1.4<br>Macrosomia: 2.0	1.4-1.9<br>1.2-2.2<br>1.2-1.7<br>1.8-2.3

						Induction of labour: 3.0<br>Caesarean section: 1.7	2.7–3.4<br>1.6–1.9
Catherine R. Chamberlain (53)	Infants of GDM mothers	Infants of non-GDM mothers		Pregnancy outcomes	OR	Predominant breastfeeding: 0.32	0.27-0.38
Phillipa B. Sharpe (92)	Infants of mother with PEDM/GDM/IGT	General population		Pregnancy outcomes	RR	Congenital anomalies:<br>PEDM: 2.01<br>GDM/IGT: 1.19	 1.66-2.44<br>1.08-1.31
A. W. Shand (19)	Pregnant women with GDM	Pregnant women without GDM		Pregnancy outcomes	OR	Gestational hypertension: 1.74<br>Induction of labour: 1.54<br>Caesarean section before labour: 1.77<br>Caesarean section before labour: 1.48	1.64-1.85<br>1.49-1.60<br>1.70-1.85<br>1.41-1.55

** Results for all-cause of death*

CHD Coronary heart disease, *CVD* Cardiovascular disease, *GDM* Gestational diabetes, *HR* Hazard ratio, *IGT* Impaired glucose tolerance, *MI* Myocardial infarction, *PAD* Peripheral arterial disease, *PEDM* Pre-existing Diabetes Mellitus, *PY* person-years, *OR* Odds ratio, *RR* Relative risk, S.aureus Staphylococcus aureus, *SMRs* Standardised mortality ratio

Table 1b: Detailed characteristics of studies investigating the relationship between diabetes and other diseases

Study	Target group	Comparator	Other diseases/health conditions	Outcome	Main outcome measure	Result	95% CI
D. G. Bruce (64)	People with diabetes and depression	People with diabetes, without depression	Depression	Mortality	HR	1.21	0.95-1.55
Jessica L. Harding (16)	People with diabetes	General population	Cancer	Cancer incidence<br>Mortality	SIRs<br>SMRs	SIRs for T1DM<br>Males: 1.02<br>Females: 1.10<br>SIRs for T2DM<br>Male: 1.08<br>Females: 1.22<br>SMRs for T1DM<br>Males: 1.19<br>Females: 1.32<br>SMRs for T2DM<br>Males: 1.03<br>Females: 1.32	 0.96-1.09<br>1.04-1.17<br><br>1.07-1.09<br>1.20-1.23<br><br>1.07-1.33<br>1.17-1.49<br><br>1.01-1.04<br>1.17-1.49
David G. Bruce (73)	People with diabetes		Dementia	Mortality	Cause-specific HR	Diabetes duration (1 year increment): 1.02	1.01-1.04
Wendy A. Davis (78)	People with diabetes	People without diabetes	Dementia	Dementia incidence<br>Mortality	Subdistribution HR	1.18	1.00, 1.39
Janine M. Duke (109)	Burn patients	Non-burn trauma patients<br>Non-injured people	Burn	Diabetes-related hospitalisation	IRR	2.21<br>1.63	1.80–2.72<br>1.24–2.14

Dianna Josephine Magliano (10)	People with diabetes	General population	Infection	Mortality	SMRs	Infection-related A-B<br>Type 1: 4.42<br>Type 2: 1.47<br>Pneumonia<br>Type 1: 5.77<br>Type 2: 1.22<br>Septicemia<br>Type 1: 9.86<br>Type 2: 1.87<br>Osteomyelitis<br>Type 1: 29.56<br>Type 2: 3.28	 3.68–5.34<br>1.42–1.53<br><br>4.25–7.83<br>1.17–1.27<br><br>7.20–13.50<br>1.76–1.97<br><br>14.71–59.1<br>2.78–3.88
Ming Li (24)	Indigenous patients with diabetes	Indigenous patients without diabetes	Infection	Hospitalisation for infection	RR	2.1	1.6-2.8
T. M. E. Davis (60)	People with diabetes	People without diabetes	Pancreatitis	Hospitalisation for pancreatitis	HR	1.73	1.06–2.83
Dianna J Magliano (79)	People with diabetes	People without diabetes	Cancer	Cancer incidence	IRR	All-cause cancer: 1.26<br>Colorectal cancer: 1.36<br>Prostate cancer: 0.83<br>Breast cancer: 0.86<br>Pancreas cancer: 2.26	1.11-1.43<br>1.01-1.82<br>0.59-1.14<br>0.52-1.36<br>1.20-4.10
Emma J. Hamilton (61)	People with diabetes	People without diabetes	Infection	Hospitalisation for infection	IRR	All infection: 2.13<br>Pneumonia: 1.86<br>Cellulitis: 2.45<br>Septicemia/bacteremia: 2.08	1.88–2.42<br>1.55–2.21<br>1.92–3.12<br>1.41–3.04
M. H. B. Zakaria (86)	People with diabetes	People without diabetes	Tendon rupture	Hospitalisation for tendon rupture	IRR	1.44	1.10–1.87
Janine M. Duke (87)	Burn patients	Non-injury people	Burn	Diabetes-related hospitalisation	IRR	2.21	1.80–2.72

Emma Hamilton (76)	People with diabetes	People without diabetes	Hip Fracture	Hip fracture hospitalisation	IRR<br>Subdistribution HR	1.33<br>1.21	1.05-1.68<br>0.96-1.52
Qun Mai (97)	MHCs	Non-MHCs	Mental illness	Diabetes prevalence<br>Diabetes-related hospitalisation<br>Mortality	OR<br>RR	1.4<br>1.2<br>1.43	1.36-1.43<br>1.17-1.24<br>1.35-1.52
Julie Hart (54)	People with diabetes		Infection	Prevalence of *S.aureus* carriage	%	39.10	
M. N. Cooper (26)	People with diabetes	People without diabetes	Psychiatric disorders	Psychiatric disorders incidence	HR	2.3	1.9-2.7
W. A. Davis (115)	People with diabetes	People without diabetes	Suicide	Mortality	HR	1.16	0.38-3.51
Claudia Caroline Dobler (71)	People with diabetes	People without diabetes	Tuberculosis	TB incidence	RR	1.48	1.04 to 2.10
Wendy Angela Davis (98)	People with diabetes	People without diabetes	Schizophrenia	Mortality	HR	5.86	2.44-14.11

HR Hazard ratio, *IRR* Incidence risk ratio, *MHCs* Mental Health Clients, *RR* Relative risk, *SIRs* Standardised incidence, *SMRs* Standardised mortality ratios, *TB* Tuberculosis

Table 2: Detailed characteristics of studies investigating risk factor for diabetes and its complications

Study	Target group	Comparator	Complication	Main outcome measure	Type of factor	Result	95% CI
Mendel Baba (50)	People with diabetes		Foot ulcer	HR	Multiple	Retinopathy: 3.86<br>Cerebrovascular disease: 3.76<br>Intermitten claudication: 2.77<br>Peripheral sensory neuropathy: 2.24<br>eGFR <60 mL/min/1.73 m2: 2.12<br>Peripheral arterial disease: 1.85<br>HbA1c (for a 1.0% increase): 1.22	2.26-6.59<br>1.97-7.19<br>1.52-5.04<br>1.35-3.71<br>1.30-3.51<br>1.10-3.13<br>1.07-1.40
Xiaotong Han (55)	People with diabetes who formerly/currently smoke	People with diabetes who never smoke	Cataract	RR	Lifestyle	Former smokers: 0.96<br>Current smokers: 0.82	0.84-1.10<br>0.64-1.05
Changfan Wu (56)	People with diabetes with ≥ 5 PA sessions/week	People with diabetes with < 5 PA sessions/week	Cataract	HR	Lifestyle	PA (session/week):<br>≥5–9: 1.01<br>≥9–14: 0.88<br>≥14: 0.81	 0.86-1.19<br>0.73-1.06<br>0.67-0.99
Timothy M. E. Davis (43)	People with diabetes		Hypoglycemia	RR	Multiple	Time on insulin (increase of 1 year): 1.33<br>History of severe hypoglycemia: 5.66<br>eGFR <60 ml/min per 1.73 m2: 2.39<br>Peripheral neuropathy: 2.44<br>Educational attainment higher than primary level: 2.34	1.15-1.53<br>2.21-14.50<br>1.37-4.15<br>1.33-4.47<br>1.09-5.04
Xixi Yan (45)	People with diabetes		Retinopathy	HR	Lifestyle	Higher consumption of cheese: 0.58<br>Higher consumption of whole-meal bread: 0.64	0.41-0.83<br>0.46-0.89
Changfan Wu (68)	People with diabetes		Cataract	HR	Lifestyle	For women:<br>Increasing consumption of red meat: 1.39<br>Increasing consumption of poultry: 1.40	 1.00-1.93<br>1.05-1.87

Jocelyn J. Drinkwater (75)	People with diabetes		Cataract	Sub-distributional HR	Multiple	Age at study entry (increase of 1 year): 0.069<br>Diabetes duration (increase of 1 year): 0.773<br>Serum HDL-C (increase of 0.1 mmol/L): 0.077<br>Ln (serum triglycerides) (mmol/L): 0.473<br>Severe hypoglycemic episode in past year: 0.794<br>Asian ethnicity: 0.696<br>Southern European ethnicity: 0.342	0.038-0.100<br>0.392-1.154<br>0.043-0.110<br>0.227-0.720<br>0.349-1.238<br>0.209-1.183<br>0.043-0.641
Matthew N. Cooper (52)	People with diabetes		Vascular complications	HR	Multiple	Poor glycaemic control during paediatric management: 8.4<br>Women: 4.35	2.0-34.7<br>1.47-12.84
Mary White (113)	People with diabetes		Retinopathy	OR	Clinical	Higher mean paediatric HbA1c levels: 2.9<br>Higher mean adult HbA1c levels: 2.1<br>Diabetes duration (increase of 1 year): 1.3	1.9-4.3<br>1.4-3.1<br>1.2-1.5
David G. Bruce (100)	People with diabetes		MI	HR	Socio-demographic	Maternal Family History of Diabetes in female: 0.45	0.26-0.76
T. M. E. Davis (118)	People with diabetes, without history of stroke at baseline, developed stroke during follow-up (3)	People with diabetes, without (1)/with history of stroke (2)	Stroke	Mean ± SD	Clinical	Serum HDL-cholesterol (1): 1.26 ± 0.42<br>Group (2): 1.28 ± 0.45<br>Group (3): 0.69 ± 0.17	
W. A. Davis (46)	People with diabetes		Amputation	HR	Multiple	History of cerebrovascular disease: 5.45<br>HbA1c (increase of 1%): 1.30<br>Any retinopathy present: 2.99<br>Neuropathy present: 2.65<br>Ln (urinary albumin:creatinine ratio): 1.34<br>Ankle/brachial index < 0.90: 2.21<br>Foot ulcer present (either foot): 5.56	2.51-11.85<br>1.10-1.54<br>1.47-6.08<br>1.30-5.44<br>1.07-1.66<br>1.11-4.42<br>1.24-25.01
Melissa Gillett (80)	People with diabetes		Stroke	HR	Multiple	Carotid bruit: 6.7	3.0-14.9

Kirsten E. Peters (103)	People with diabetes		CVD	HR	Clinical	Log e (serum adiponectin): 0.79<br>The ADIPOQ variant rs12495941: 0.64<br>The ADIPOQ variant rs1648707: 2.05	0.65-0.98<br>0.44-0.94<br>1.37-3.06
Amanda Ampt (58)	People with diabetes		Diabetic ketoacidosis	OR	Multiple	Female: 1.49<br>Residential area of inner regional: 1.26<br>Outer regional/remote areas: 1.28<br>Residential area of socioeconomic disadvantage: 3.00	1.24-1.79<br>1.02-1.56<br>0.89-1.85<br>2.14-4.20
Jocelyn J. Drinkwater (94)	People with diabetes		Stroke, myocardial infarction	HR	Clinical	Moderate NPDR or worse: 2.55	1.19-5.47
Timothy M E Davis (33)	People with diabetes		Diabetic ketoacidosis	HR	Clinical	Ln(serum C- peptide (pmol/L)): 0.44<br>HbA1c (increase of 1% or 7 mmol/mol): 1.76<br>Secondary diabetes: 22.1	0.33-0.59<br>1.41-2.19<br>4.82-101
Louisa Sukkar (47)	People with diabetes		Chronic kidney disease	HR	Multiple	Older age: 1.23<br>Remoteness of residence: 1.36<br>Obesity: 1.44<br>Hypertension: 1.52<br>Coronary heart disease: 1.13<br>Cancer: 1.30<br>Depression/anxiety: 1.14	1.19–1.26<br>1.17-1.58<br>1.16–1.80<br>1.33–1.73<br>1.02–1.24<br>1.14–1.50<br>1.01–1.27
M. Begum (2)	Children born by caesarean	Children born by vaginal delivery		HR	Perinatal	Caesarean: 1.05	0.86-1.28
Aveni Haynes (48)	People with diabetes and maternal smoking	People with diabetes, without maternal smoking		HR	Lifestyle	Maternal smoking: 0.76	0.54-1.08
A. Z. Khambalia (49)	Pregnant women			RR	Multiple	GDM in the first pregnancy: 21.33	19.90-22.86

A. Haynes (12)	People with diabetes			IRR	Perinatal	Mother with pre-existing diabetes: 4.74	2.93-7.66
						Mother with Caucasian ethnicity: 3.73	2.61-5.34
						Increasing birth weight: 1.13	1.04-1.23
C. S. Algert (117)	People with diabetes			Crude RR	Perinatal	Maternal T1DM: 6.33	2.62-15.3
						Caesarean section: 1.3	1.01-1.69
						Increasing birth weight: 1.09	0.97-1.22
Valerie Burke (93)	Aboriginal people			HR	Lifestyle	Waist girth: 1.08	1.04-1.13
						Smoking: 2.05	1.23-3.39
						Eating processed meats > 4 times/month: 1.58	1.05-2.40
						Lower alcohol intake: 0.69	0.49-0.99
						Preferring wine: 0.13	0.02-0.97
						Eating bush meats > 4 times/month: 0.34	0.13-0.90
Sandra K. Campbell (30)	Indigenous pregnant women			PR	Multiple	Metabolic syndrome: 3.5	1.54-8.00
						Hyper-triglyceridaemic waist: 3.23	1.53-6.84
						Fasting glucose: 1.55	1.35-1.76
						Waist circumference: 1.04	1.01-1.06
						Systolic blood pressure: 1.03	1.01-1.05
						Diastolic blood pressure: 1.03	1.00-1.07
Catherine R. Chamberlain (57)	Indigenous pregnant women	Non-indigenous pregnant women		HR	Multiple	Indigenous: 4.55	2.63-7.88
						BMI 25-29: 3.16	1.01-9.86
						Partially breastfeeding at discharge: 2.34	1.23-4.47
						GDM diagnosis prior to 17 weeks gestation: 5.38	2.56-11.37
Mumtaz Begum (4)	People with diabetes and maternal smoking	People with diabetes, without maternal smoking		HR	Lifestyle	Maternal smoking: 0.84	0.67-1.08
Hongmei Zhang (108)	Australians aged ≥ 45			HR	Multiple	Older age: 1.06	1.05-1.07
						Female: 0.61	0.58-0.64
						Family history of diabetes: 1.65	1.57-1.73
						Personal history of CVD: 1.10	1.04-1.16
						Personal history of hypertension: 1.75	1.67-1.84
						Higher body mass index (≥ 40.0): 6.65	5.93-7.45

					Current smoker: 1.54<br>Long sleeping hours (≥ 10): 1.15	1.41-1.67<br>1.06-1.24
Mark Jones (38)	Elderly women with statin use	Elderly women without statin use		HR	Statin use: 1.3	1.04-1.70
Timothy M. E. Davis (91)	Aboriginal people with diabetes	Anglo-Celt people with diabetes	Microvascular (Retinopathy)	Prevalence	Phase I: 28.7%/13.5%<br>Phase II: 33.0%/19.2%	
Mumtaz Begum (21)	Children			Incidence rate of T1DM	23.0 per 100 000 PY	
Julia R. Kurowski (8)	People with/without diabetes having CVD		Lower amputations	Recurrent lower amputation rates	T2DM: +3.5%/year	+1.3, +5.7

CVD Cardiovascular disease, *GDM* Gestational diabetes, *HR* Hazard ratio, *IRR* Incidence risk ratio, NPDR Non-proliferative diabetic retinopathy, PA Physical activity, *PY* person-years, *RR* Relative risk

1. Comino EJ, Tran DT, Haas M, Flack J, Jalaludin B, Jorm L, et al. Validating self-report of diabetes use by participants in the 45 and Up Study: a record linkage study. BMC health services research. 2013;13:481.
2. Begum M, Pilkington R, Chittleborough C, Lynch J, Penno M, Smithers L. Caesarean section and risk of type 1 diabetes: whole-of-population study. Diabetic Medicine.
3. Dennis S, Taggart J, Yu H, Jalaludin B, Harris MF, Liaw ST. Linking observational data from general practice, hospital admissions and diabetes clinic databases: can it be used to predict hospital admission? BMC health services research. 2019;19(1):526.
4. Begum M, Pilkington RM, Chittleborough CR, Lynch JW, Penno M, Smithers LG. Effect of maternal smoking during pregnancy on childhood type 1 diabetes: a whole-of-population study. Diabetologia. 2020;63(6):1162-73.
5. Ha NT, Harris M, Preen D, Moorin R. Time protective effect of contact with a general practitioner and its association with diabetes-related hospitalisations: a cohort study using the 45 and Up Study data in Australia. BMJ open. 2020;10(4):e032790.

6. Comino EJ, Islam MDF, Tran DT, Jorm L, Flack J, Jalaludin B, et al. Association of processes of primary care and hospitalisation for people with diabetes: A record linkage study. Diabetes Research and Clinical Practice. 2015;108(2):296-305.
7. Nedkoff L, Knuiman M, Hung J, Sanfilippo FM, Katzenellenbogen JM, Briffa TG. Concordance between administrative health data and medical records for diabetes status in coronary heart disease patients: a retrospective linked data study. BMC Medical Research Methodology. 2013;13:121.
8. Kurowski JR, Nedkoff L, Schoen DE, Knuiman M, Norman PE, Briffa TG. Temporal trends in initial and recurrent lower extremity amputations in people with and without diabetes in Western Australia from 2000 to 2010. Diabetes Research and Clinical Practice. 2015;108(2):280-7.
9. Harding JL, Shaw JE, Peeters A, Davidson S, Magliano DJ. Age-specific trends from 2000–2011 in all-cause and cause-specific mortality in type 1 and type 2 diabetes: a cohort study of more than one million people. Diabetes Care. 2016;39(6):1018-26.
10. Magliano DJ, Harding JL, Cohen K, Huxley RR, Davis WA, Shaw JE. Excess risk of dying from infectious causes in those with type 1 and type 2 diabetes. Diabetes Care. 2015;38(7):1274-80.
11. Harding JL, Shaw JE, Peeters A, Guiver T, Davidson S, Magliano DJ. Mortality Trends Among People With Type 1 and Type 2 Diabetes in Australia: 1997-2010. Diabetes Care. 2014;37(9):2579-86.
12. Haynes A, Bower C, Bulsara MK, Finn J, Jones TW, Davis EA. Perinatal risk factors for childhood Type 1 diabetes in Western Australia—a population-based study (1980–2002). Diabetic Medicine. 2007;24(5):564-70.
13. Morton JI, Ilomäki J, Magliano DJ, Shaw JE. The association of socioeconomic disadvantage and remoteness with receipt of type 2 diabetes medications in Australia: a nationwide registry study. Diabetologia. 2021;64(2):349-60.
14. Nedkoff L, Knuiman M, Hung J, Briffa TG. Long-term all-cause and cardiovascular mortality following incident myocardial infarction in men and women with and without diabetes: Temporal trends from 1998 to 2009. European Journal of Preventive Cardiology. 2016;23(12):1273-81.
15. Gibson OR, Segal L, McDermott RA. A simple diabetes vascular severity staging instrument and its application to a Torres Strait Islander and Aboriginal adult cohort of north Australia. BMC health services research. 2012;12:185.
16. Harding JL, Shaw JE, Peeters A, Cartensen B, Magliano DJ. Cancer Risk Among People With Type 1 and Type 2 Diabetes: Disentangling True Associations, Detection Bias, and Reverse Causation. Diabetes Care. 2015;38(2):264-70.
17. Stone CA, McLachlan KA, Halliday JL, Wein P, Tippett C. Gestational diabetes in Victoria in 1996: Incidence, risk factors and outcomes. Medical Journal of Australia. 2002;177(9):486-91.
18. Stewart Williams J, Ling R, Searles AM, Doran CM, Byles J. Identification of higher hospital costs and more frequent admissions among mid-aged Australian women who self-report diabetes mellitus. Maturitas. 2016;90:58-63.
19. Shand AW, Bell JC, McElduff A, Morris J, Roberts CL. Outcomes of pregnancies in women with pre-gestational diabetes mellitus and gestational diabetes mellitus; a population-based study in New South Wales, Australia, 1998–2002. Diabetic Medicine. 2008;25(6):708-15.
20. Zhao Y, Connors C, Lee AH, Liang W. Relationship between primary care visits and hospital admissions in remote indigenous patients with diabetes: A multivariate spline regression model. Diabetes Research and Clinical Practice. 2015;108(1):106-12.
21. Begum M, Chittleborough C, Pilkington R, Mittinty M, Lynch J, Penno M, et al. Incidence of type 1 diabetes by socio-demographic characteristics among South Australian children: Whole-of-population study. Journal of Paediatrics and Child Health. 2020;56(12):1952-8.

22. Al-Saeed AH, Constantino MI, Molyneaux L, D'Souza M, Limacher-Gisler F, Luo C, et al. An inverse relationship between age of type 2 diabetes onset and complication risk and mortality: the impact of youth-onset type 2 diabetes. Diabetes Care. 2016;39(5):823-9.
23. Alharbi TJ, Constantino MI, Molyneaux L, Wu T, Twigg SM, Yue DK, et al. Ethnic specific differences in survival of patients with type 2 diabetes: Analysis of data collected from an Australian multi-ethnic cohort over a 25 year period. Diabetes Research and Clinical Practice. 2015;107(1):130-8.
24. Li M, McDermott R. High absolute risk of severe infections among Indigenous adults in rural northern Australia is amplified by diabetes - A 7 year follow up study. Journal of diabetes and its complications. 2016;30(6):1069-73.
25. Comino EJ, Harris MF, Islam MD, Tran DT, Jalaludin B, Jorm L, et al. Impact of diabetes on hospital admission and length of stay among a general population aged 45 year or more: a record linkage study. BMC health services research. 2015;15:12.
26. Cooper MN, Lin A, Alvares GA, de Klerk NH, Jones TW, Davis EA. Psychiatric disorders during early adulthood in those with childhood onset type 1 diabetes: Rates and clinical risk factors from population-based follow-up. Pediatric Diabetes. 2017;18(7):599-606.
27. Hayes AJ, Davis WA, Davis TM, Clarke PM. Adapting and validating diabetes simulation models across settings: Accounting for mortality differences using administrative data. Journal of Diabetes and its Complications. 2013;27(4):351-6.
28. Zhang J, Donald M, Baxter KA, Ware RS, Burridge L, Russell AW, et al. Impact of an integrated model of care on potentially preventable hospitalizations for people with Type 2 diabetes mellitus. Diabetic Medicine. 2015;32(7):872-80.
29. Youens D, Preen DB, Harris MN, Moorin RE. The importance of historical residential address information in longitudinal studies using administrative health data. International Journal of Epidemiology. 2018;47(1):69-80.
30. Campbell SK, Lynch J, Esterman A, McDermott R. Pre-pregnancy predictors of diabetes in pregnancy among Aboriginal and Torres Strait Islander women in North Queensland, Australia. Maternal and child health journal. 2012;16(6):1284-92.
31. Clarke P, Leal J, Kelman C, Smith M, Colagiuri S. Estimating the cost of complications of diabetes in Australia using administrative health-care data. Value in Health. 2008;11(2):199-206.
32. Moorin RE, Youens D, Preen DB, Harris M, Wright CM. Association between continuity of provider-adjusted regularity of general practitioner contact and unplanned diabetes-related hospitalisation: A data linkage study in New South Wales, Australia, using the 45 and Up Study cohort. BMJ Open. 2019;9 (6) (no pagination)(e027158).
33. Davis TM, Davis W. Incidence and associates of diabetic ketoacidosis in a community-based cohort: the Fremantle Diabetes Study Phase II. BMJ Open Diabetes Research and Care. 2020;8(1):e000983.
34. Glatthaar C, Whittall DE, Welborn TA, Gibson MJ, Brooks BH, Ryan MM, et al. Diabetes in Western Australian children: descriptive epidemiology. Medical Journal of Australia. 1988;148(3):117-23.
35. Clarke P, Kelman C, Colagiuri S. Factors influencing the cost of hospital care for people with diabetes in Australia. Journal of diabetes and its complications. 2006;20(6):349-55.
36. Huo L, Magliano DJ, Ranciere F, Harding JL, Nanayakkara N, Shaw JE, et al. Impact of age at diagnosis and duration of type 2 diabetes on mortality in Australia 1997-2011. Diabetologia. 2018;61(5):1055-63.
37. Huo L, Harding JL, Peeters A, Shaw JE, Magliano DJ. Life expectancy of type 1 diabetic patients during 1997-2010: a national Australian registry-based cohort study. Diabetologia. 2016;59(6):1177-85.

38. Jones M, Tett S, Peeters G, Mishra GD, Dobson A. New-Onset Diabetes After Statin Exposure in Elderly Women: The Australian Longitudinal Study on Women's Health. Drugs & Aging. 2017;34(3):203-9.
39. Magliano DJ, Cohen K, Harding JL, Shaw JE. Residential distance from major urban areas, diabetes and cardiovascular mortality in Australia. Diabetes Research and Clinical Practice. 2015;109(2):271-8.
40. Boyle DIR, Versace VL, Dunbar JA, Scheil W, Janus E, Oats JJN, et al. Results of the first recorded evaluation of a national gestational diabetes mellitus register: Challenges in screening, registration, and follow-up for diabetes risk. PloS one. 2018;13 (8) (no pagination)(e0200832).
41. Nedkoff L, Knuiman M, Hung J, Briffa TG. Improving 30-day case fatality after incident myocardial infarction in people with diabetes between 1998 and 2010. Heart. 2015;101(16):1318-24.
42. De Klerk NH, Armstrong BK. Admission to hospital for road trauma in patients with diabetes mellitus. Journal of Epidemiology and Community Health. 1983;37(3):232-7.
43. Davis TME, Brown SGA, Jacobs IG, Bulsara M, Bruce DG, Davis WA. Determinants of Severe Hypoglycemia Complicating Type 2 Diabetes: The Fremantle Diabetes Study. The Journal of Clinical Endocrinology & Metabolism. 2010;95(5):2240-7.
44. Tran DT, Jorm LR, Havard A, Harris MF, Comino EJ. Variation in the use of primary care services for diabetes management according to country of birth and geography among older Australians. Primary care diabetes. 2016;10(1):66-74.
45. Yan X, Han X, Wu C, Keel S, Shang X, Zhang L, et al. Does daily dietary intake affect diabetic retinopathy progression? 10-year results from the 45 and Up Study. British Journal of Ophthalmology. 2019.
46. Davis WA, Norman PE, Bruce DG, Davis TME. Predictors, consequences and costs of diabetes-related lower extremity amputation complicating type 2 diabetes: The Fremantle Diabetes Study. Diabetologia. 2006;49(11):2634-41.
47. Sukkar L, Kang A, Hockham C, Young T, Jun M, Foote C, et al. Incidence and associations of chronic kidney disease in community participants with diabetes: a 5-year prospective analysis of the EXTEND45 study. Diabetes care. 2020;43(5):982-90.
48. Haynes A, Cooper MN, Bower C, Jones TW, Davis EA. Maternal smoking during pregnancy and the risk of childhood type 1 diabetes in Western Australia. Diabetologia. 2014;57(3):469-72.
49. Khambalia AZ, Ford JB, Nassar N, Shand AW, McElduff A, Roberts CL. Occurrence and recurrence of diabetes in pregnancy. Diabetic Medicine. 2013;30(4):452-6.
50. Baba M, Davis WA, Davis TM. A longitudinal study of foot ulceration and its risk factors in community-based patients with type 2 diabetes: the Fremantle Diabetes Study. Diabetes Research & Clinical Practice. 2014;106(1):42-9.
51. Davis WA, Knuiman MW, Hendrie D, Davis TME. Determinants of Diabetes-Attributable Non-Blood Glucose-Lowering Medication Costs in Type 2 Diabetes: The Fremantle Diabetes Study. Diabetes Care. 2005;28(2):329-36.
52. Cooper MN, de Bock MI, Carter KW, de Klerk NH, Jones TW, Davis EA. Incidence of and risk factors for hospitalisations due to vascular complications: A population-based type 1 diabetes cohort (n = 1316) followed into early adulthood. Journal of diabetes and its complications. 2017;31(5):843-9.
53. Chamberlain CR, Wilson AN, Amir LH, O'Dea K, Campbell S, Leonard D, et al. Low rates of predominant breastfeeding in hospital after gestational diabetes, particularly among Indigenous women in Australia. Australian and New Zealand journal of public health. 2017;41(2):144-50.

54. Hart J, Hamilton EJ, Makepeace A, Davis WA, Latkovic E, Lim EM, et al. Prevalence, risk factors and sequelae of Staphylococcus aureus carriage in diabetes: the Fremantle Diabetes Study Phase II. Journal of Diabetes & its Complications. 2015;29(8):1092-7.
55. Han X, Wu C, Yan X, Keel S, Shang X, Zhang L, et al. Are smoking intensity and cessation related to cataract surgical risk in diabetic patients? Findings from the 45 and Up Study. Eye. 2019.
56. Wu C, Han X, Yan X, Shang X, Zhang L, He M. Associations between physical activity and cataract treated surgically in patients with diabetes: Findings from the 45 and Up Study. British Journal of Ophthalmology. 2019;103(8):1099-105.
57. Chamberlain CR, Oldenburg B, Wilson AN, Eades SJ, O'Dea K, Oats JJ, et al. Type 2 diabetes after gestational diabetes: greater than fourfold risk among Indigenous compared with non-Indigenous Australian women. Diabetes/Metabolism Research Reviews. 2016;32(2):217-27.
58. Ampt A, van Gemert T, Craig ME, Donaghue KC, Lain SB, Nassar N. Using population data to understand the epidemiology and risk factors for diabetic ketoacidosis in Australian children with type 1 diabetes. Pediatric Diabetes. 2019;20(7):901-8.
59. Hayes AJ, Leal J, Kelman CW, Clarke PM. Risk equations to predict life expectancy of people with Type 2 diabetes mellitus following major complications: a study from Western Australia. Diabetic Medicine. 2011;28(4):428-35.
60. Davis TM, Drinkwater J, Davis WA. Incidence and precipitants of hospitalization for pancreatitis in people with diabetes: the Fremantle Diabetes Study. Diabetic Medicine. 2014;31(8):913-9.
61. Hamilton EJ, Martin N, Makepeace A, Sillars BA, Davis WA, Davis TME. Incidence and Predictors of Hospitalization for Bacterial Infection in Community-Based Patients with Type 2 Diabetes: The Fremantle Diabetes Study. PLoS ONE. 2013;8(3):e60502.
62. Youens D, Harris M, Robinson S, Preen DB, Moorin RE. Regularity of contact with GPs: Measurement approaches to improve valid associations with hospitalization. Family practice. 2019;36(5):650-6.
63. Davis WA, Bruce DG, Davis TM. Does self-monitoring of blood glucose improve outcome in type 2 diabetes? The Fremantle Diabetes Study. Diabetologia. 2007;50(3):510-5.
64. Bruce DG, Davis WA, Starkstein SE, Davis TME. A prospective study of depression and mortality in patients with type 2 diabetes: the Fremantle Diabetes Study. Diabetologia. 2005;48(12):2532-9.
65. Chamberlain C, Fredericks B, McLean A, Oldenburg B, Mein J, Wolfe R. Associations with low rates of postpartum glucose screening after gestational diabetes among Indigenous and non-Indigenous Australian women. Australian and New Zealand journal of public health. 2015;39(1):69-76.
66. Davis WA, Lewin G, Davis TM, Bruce DG. Determinants and costs of community nursing in patients with type 2 diabetes from a community-based observational study: the Fremantle Diabetes Study. International Journal of Nursing Studies. 2013;50(9):1166-71.
67. Chamberlain C, Fredericks B, McLean A, Davis B, Eades S, Stewart K, et al. Gestational diabetes mellitus in Far North Queensland, Australia, 2004 to 2010: midwives' perinatal data most accurate source. Australian and New Zealand journal of public health. 2013;37(6):556-61.
68. Wu C, Han X, Yan X, Keel S, Shang X, Zhang L, et al. Impact of Diet on the Incidence of Cataract Surgery among Diabetic Patients: Findings from the 45 and Up Study. Current Eye Research. 2019;44(4):385-92.
69. Abdelhamid YA, Plummer MP, Finnis ME, Biradar V, Bihari S, Kar P, et al. Long-term mortality of critically ill patients with diabetes who survive admission to the intensive care unit. Critical Care and Resuscitation. 2017;19(4):303-9.

70. Chamberlain C, McLean A, Oats J, Oldenburg B, Eades S, Sinha A, et al. Low rates of postpartum glucose screening among indigenous and non-indigenous women in Australia with gestational diabetes. Maternal and child health journal. 2015;19(3):651-63.
71. Dobler CC, Flack JR, Marks GB. Risk of tuberculosis among people with diabetes mellitus: An Australian nationwide cohort study. BMJ Open. 2012;2 (1) (no pagination)(000666).
72. Ha NT, Harris M, Preen D, Robinson S, Moorin R. A time-duration measure of continuity of care to optimise utilisation of primary health care: a threshold effects approach among people with diabetes. BMC health services research. 2019;19(1):276.
73. Bruce DG, Davis TME, Davis WA. Dementia complicating type 2 diabetes and the influence of premature mortality: the Fremantle Diabetes Study. Acta diabetologica. 2019;56(7):767-76.
74. Ha NT, Harris M, Preen D, Robinson S, Moorin R. Identifying patterns of general practitioner service utilisation and their relationship with potentially preventable hospitalisations in people with diabetes: The utility of a cluster analysis approach. Diabetes Research and Clinical Practice. 2018;138:201-10.
75. Drinkwater JJ, Davis TME, Turner AW, Bruce DG, Davis WA. Incidence and determinants of intraocular lens implantation in type 2 diabetes: The fremantle diabetes study phase II. Diabetes Care. 2019;42(2):288-96.
76. Hamilton E, Davis WA, Bruce DG, Davis TME. Influence of premature mortality on the link between type 2 diabetes and hip fracture: The fremantle diabetes study. Journal of Clinical Endocrinology and Metabolism. 2017;102(2):551-9.
77. Ha NT, Harris M, Robinson S, Preen D, Moorin R. Stratification strategy for evaluating the influence of diabetes complication severity index on the risk of hospitalization: a record linkage data in Western Australia. Journal of diabetes and its complications. 2017;31(7):1175-80.
78. Davis WA, Zilkens RR, Starkstein SE, Davis TM, Bruce DG. Dementia onset, incidence and risk in type 2 diabetes: a matched cohort study with the Fremantle Diabetes Study Phase I. Diabetologia. 2017;60(1):89-97.
79. Magliano DJ, Davis WA, Shaw JE, Bruce DG, Davis TME. Incidence and predictors of all-cause and site-specific cancer in type 2 diabetes: the Fremantle Diabetes Study. European Journal of Endocrinology. 2012;167(4):589-99.
80. Gillett M, Davis WA, Jackson D, Bruce DG, Davis TME. Prospective Evaluation of Carotid Bruit as a Predictor of First Stroke in Type 2 Diabetes. Stroke. 2003;34(9):2145-51.
81. Gibson AA, Humphries J, Gillies M, Nassar N, Colagiuri S. Adherence to eye examination guidelines among individuals with diabetes: an analysis of linked health data. Clinical & Experimental Ophthalmology. 2020;48(9):1229-38.
82. Davis WA, Colagiuri S, Davis TME. Comparison of the Framingham and United Kingdom Prospective Diabetes Study cardiovascular risk equations in Australian patients with type 2 diabetes from the Fremantle Diabetes Study. Medical Journal of Australia. 2009;190(4):180-4.
83. Brameld KJ, Ward A, Gavin AL, Holman CD. Health outcomes in people with type 2 diabetes. A record linkage study. Australian Family Physician. 2002;31(8):775-8, 82.
84. Afzali HHA, Gray J, Beilby J, Holton C, Banham D, Karnon J. A risk-adjusted economic evaluation of alternative models of involvement of practice nurses in management of type 2 diabetes. Diabetic Medicine. 2013;30(7):855-63.
85. Kamber N, Davis WA, Bruce DG, Davis TM. Metformin and lactic acidosis in an Australian community setting: the Fremantle Diabetes Study. Medical Journal of Australia. 2008;188(8):446-9.

86. Zakaria MHB, Davis WA, Davis TME. Incidence and predictors of hospitalization for tendon rupture in Type 2 diabetes: the Fremantle Diabetes Study. Diabetic Medicine. 2014;31(4):425-30.
87. Duke JM, Randall SM, Fear MW, Boyd JH, O'Halloran E, Rea S, et al. Increased admissions for diabetes mellitus after burn. Burns. 2016;42(8):1734-9.
88. Cheung NW, Jiang S, Athayde N. Impact of the IADPSG criteria for gestational diabetes, and of obesity, on pregnancy outcomes. Australian and New Zealand Journal of Obstetrics and Gynaecology. 2018;58(5):553-9.
89. Davis TME, Fortun P, Mulder J, Davis WA, Bruce DG. Silent myocardial infarction and its prognosis in a community-based cohort of Type 2 diabetic patients: the Fremantle Diabetes Study. Diabetologia. 2004;47(3):395-9.
90. Abell SK, Teede HJ. The IADPSG diagnostic criteria identify women with increased risk of adverse pregnancy outcomes in Victoria. Australian & New Zealand Journal of Obstetrics & Gynaecology. 2017;57(5):564-8.
91. Davis TM, Hunt K, McAullay D, Chubb SA, Sillars BA, Bruce DG, et al. Continuing disparities in cardiovascular risk factors and complications between aboriginal and Anglo-Celt Australians with type 2 diabetes: the Fremantle Diabetes Study. Diabetes care. 2012;35(10):2005-11.
92. Sharpe PB, Chan A, Haan EA, Hiller JE. Maternal diabetes and congenital anomalies in South Australia 1986-2000: A population-based cohort study. Birth Defects Research Part A - Clinical and Molecular Teratology. 2005;73(9):605-11.
93. Burke V, Zhao Y, Lee AH, Hunter E, Spargo RA, Gracey M, et al. Predictors of type 2 diabetes and diabetes-related hospitalisation in an Australian Aboriginal cohort. Diabetes Research and Clinical Practice. 2007;78(3):360-8.
94. Drinkwater JJ, Davis TM, Hellbusch V, Turner AW, Bruce DG, Davis WA. Retinopathy predicts stroke but not myocardial infarction in type 2 diabetes: the Fremantle Diabetes Study Phase II. Cardiovascular diabetology. 2020;19(1):1-11.
95. Davis TM, Davis WA. Influence of Renin-Angiotensin System Inhibitors on Lower–Respiratory Tract Infections in Type 2 Diabetes: The Fremantle Diabetes Study Phase II. Diabetes Care. 2020;43(9):2113-20.
96. Norman PE, Davis WA, Bruce DG, Davis TME. Peripheral Arterial Disease and Risk of Cardiac Death in Type 2 Diabetes: The Fremantle Diabetes Study. Diabetes Care. 2006;29(3):575-80.
97. Mai Q, Holman CD, Sanfilippo FM, Emery JD, Preen DB. Mental illness related disparities in diabetes prevalence, quality of care and outcomes: a population-based longitudinal study. BMC Medicine. 2011;9:118.
98. Davis WA, Starkstein SE, Bruce DG, Davis TM. The interactive effects of type 2 diabetes mellitus and schizophrenia on all-cause mortality: The Fremantle Diabetes Study. Journal of Diabetes & its Complications. 2015;29(8):1320-2.
99. Kelty EA, Tran D, Atkinson AA, Preen DB, Havard A. Maternal and neonatal health outcomes associated with the use of gliclazide and metformin for the treatment of diabetes in pregnancy: a record linkage study. Diabetes technology & therapeutics. 2019;17.
100. Bruce DG, Van Minnen K, Davis WA, Mudhar J, Perret M, Subawickrama DP, et al. Maternal family history of diabetes is associated with a reduced risk of cardiovascular disease in women with type 2 diabetes: the Fremantle Diabetes Study. Diabetes Care. 2010;33(7):1477-83.
101. Wubishet BL, Harris ML, Forder PM, Acharya SH, Byles JE. Predictors of 15-year survival among Australian women with diabetes from age 76-81. Diabetes Research and Clinical Practice. 2019;150:48-56.
102. Davis WA, Knuiman MW, Hendrie D, Davis TME. The obesity-driven rising costs of type 2 diabetes in Australia: projections from the Fremantle Diabetes Study. Internal Medicine Journal. 2006;36(3):155-61.

103. Peters KE, Davis WA, Beilby J, Hung J, Bruce DG, Davis TME. The relationship between circulating adiponectin, ADIPOQ variants and incident cardiovascular disease in type 2 diabetes: The Fremantle Diabetes Study. Diabetes Research and Clinical Practice. 2018;143:62-70.
104. Peters KE, Chubb SAP, Davis WA, Davis TME. The Relationship between Hypomagnesemia, Metformin Therapy and Cardiovascular Disease Complicating Type 2 Diabetes: The Fremantle Diabetes Study. PloS one. 2013;8 (9) (no pagination)(e74355).
105. Tatoulis J, Wynne R, Skillington PD, Buxton BF. Total Arterial Revascularization: A Superior Strategy for Diabetic Patients Who Require Coronary Surgery. Annals of Thoracic Surgery. 2016;102(6):1948-55.
106. Young AF, Lowe JM, Byles JE, Patterson AJ. Trends in health service use for women in Australia with diabetes. Australian and New Zealand journal of public health. 2005;29(5):422-8.
107. Comino EJ, Tran DT, Taggart JR, Liaw ST, Ruscoe W, Snow JM, et al. A preliminary study of the relationship between general practice care and hospitalisation using a diabetes register, CARDIAB. Aust Health Rev. 2013;37(2):210-7.
108. Zhang H, Rogers K, Sukkar L, Jun M, Kang A, Young T, et al. Prevalence, incidence and risk factors of diabetes in Australian adults aged ⩾ 45 years: A cohort study using linked routinely-collected data. Journal of clinical & translational endocrinology. 2020;22:100240.
109. Duke JM, Randall SM, Fear MW, Boyd JH, Rea S, Wood FM. Diabetes mellitus after injury in burn and non-burned patients: A population based retrospective cohort study. Burns. 2018;44(3):566-72.
110. Davis WA, Knuiman MW, Davis TME. An Australian cardiovascular risk equation for type 2 diabetes: the Fremantle Diabetes Study. Internal Medicine Journal. 2010;40(4):286-92.
111. Davis TME, McAullay D, Davis WA, Bruce DG. Characteristics and outcome of type 2 diabetes in urban Aboriginal people: the Fremantle Diabetes Study. Internal Medicine Journal. 2007;37(1):59-63.
112. Tan ED, Davis WA, Davis TM. Characteristics and prognosis of Asian patients with type 2 diabetes from a multi-racial Australian community: the Fremantle Diabetes Study. Internal Medicine Journal. 2013;43(10):1125-32.
113. White M, Sabin MA, Magnussen CG, O'Connell MA, Colman PG, Cameron F. Long term risk of severe retinopathy in childhood-onset type 1 diabetes: A data linkage study. Medical Journal of Australia. 2017;206(9):398-401.
114. Abell SK, Suen M, Pease A, Boyle JA, Soldatos G, Regan J, et al. Pregnancy Outcomes and Insulin Requirements in Women with Type 1 Diabetes Treated with Continuous Subcutaneous Insulin Infusion and Multiple Daily Injections: Cohort Study. Diabetes Technology and Therapeutics. 2017;19(5):280-7.
115. Davis WA, Starkstein SE, Bruce DG, Davis TME. Risk of suicide in Australian adults with diabetes: the Fremantle Diabetes Study. Internal Medicine Journal. 2015;45(9):976-80.
116. Laafira A, White SW, Griffin CJ, Graham D. Impact of the new IADPSG gestational diabetes diagnostic criteria on pregnancy outcomes in Western Australia. Australian and New Zealand Journal of Obstetrics and Gynaecology. 2016;56(1):36-41.
117. Algert CS, McElduff A, Morris JM, Roberts CL. Perinatal risk factors for early onset of Type 1 diabetes in a 2000-2005 birth cohort. Diabetic Medicine. 2009;26(12):1193-7.
118. Davis TME, Bruce DG, Davis WA. Predictors of first stroke in type 1 diabetes: the Fremantle Diabetes Study. Diabetic Medicine. 2005;22(5):551-3.
